# Kombucha-Mediated Silver Nanoparticles with Fungicidal Activity Against WHO-Priority *Candida* Pathogens: In Vitro and *Galleria mellonella* Evaluation

**DOI:** 10.3390/cimb48060634

**Published:** 2026-06-17

**Authors:** Razvan Vlad Opris, Dan Alexandru Toc, Alina Mihaela Baciu, Ioana Alina Colosi, Vlad Sever Neculicioiu, Anca Onaciu, Cristian-Silviu Moldovan, Ana-Maria Vlase, Carmen Costache, Adrian Florea

**Affiliations:** 1 Department of Cell & Molecular Biology, “Iuliu Hatieganu” University of Medicine & Pharmacy, 6 Louis Pasteur Street, 400349 Cluj-Napoca, Romania; razvan.opris@elearn.umfcluj.ro (R.V.O.);; 2 Department of Microbiology, “Iuliu Hatieganu” University of Medicine & Pharmacy, 6 Louis Pasteur Street, 400349 Cluj-Napoca, Romania; 3 Department of NanoSciences, MedFUTURE—Institute for Biomedical Research, “Iuliu Hatieganu” University of Medicine & Pharmacy, 400349 Cluj-Napoca, Romania; 4 Department of Pharmaceutical Botany, “Iuliu Hatieganu” University of Medicine & Pharmacy, 8 Victor Babeș Street, 400347 Cluj-Napoca, Romania

**Keywords:** silver nanoparticles, kombucha, *Candida*, antifungal, *Galleria mellonella*

## Abstract

Invasive candidiasis caused by drug-resistant *Candida* species represents a critical global health challenge, with few novel therapeutic scaffolds under development. Here, silver nanoparticles were synthesized using a 21-day fermented Chun Mee kombucha tea extract (K-AgNPs) and characterized by UV-Vis spectroscopy, transmission electron microscopy, nanoparticle tracking analysis, and Fourier-transform infrared spectroscopy. LC-MS/MS profiling of the kombucha substrate documented a phytochemical landscape dominated by epigallocatechin (up to 122,631 µg/mL) and epigallocatechin gallate (up to 415 µg/mL), with a progressive ~80% decline in epicatechin and concomitant increases in gallic acid and chlorogenic acid across the 21-day fermentation. K-AgNPs obtained were spherical, 19.4 nm (±7.9 nm SD) in diameter, with a surface plasmon resonance peak at 415 nm. FTIR confirmed phenolic, carboxylate, and glycosidic surface capping. Antifungal susceptibility testing against eight *Candida* species, including the WHO critical–priority pathogen *Candidozyma auris*, showed concordant minimum inhibitory and minimum fungicidal concentrations of 0.80–1.60 µg/mL, confirming fungicidal activity. In vivo evaluation in *Galleria mellonella* larvae across six infection models demonstrated that K-AgNP treatment at the species-specific MIC significantly improved larval survival versus untreated infected controls (*p* < 0.01–0.001), while nanoparticle-only groups maintained ≥98% survival, indicating negligible toxicity. Co-treatment amplified total hemocyte mobilization, and K-AgNP-only larvae maintained hemocyte viability above 96% at all time points, indistinguishable from negative controls. Together, these findings demonstrate antifungal activity of K-AgNPs across the genus *Candida* in standardized in vitro and in vivo settings and provide justification for further investigation, including head-to-head comparison against licensed antifungals and physicochemical validation of nanoparticle stability under assay conditions.

## 1. Introduction

Invasive and mucosal candidiasis remain a growing global health burden, driven by expanding at risk populations and antifungal resistance [1]. The World Health Organization Fungal Priority Pathogens List underscores this urgency by classifying *Candidozyma auris* (*Candida auris*) and *Candida albicans* among critical priority pathogens, with *Nakaseomyces glabrata* (*Candida glabrata*), *Candida tropicalis*, and *Candida parapsilosis* in the high-priority tier [2,3]. Outbreak prone and multidrug resistant *C. auris* is now documented on multiple continents, which challenges infection control and stewardship efforts [4]. Clinically, most invasive cases are due to *C. albicans*, *N. glabrata*, *C. krusei* (*Pichia kudriavzevii*), *C. parapsilosis*, and *C. tropicalis*, while *C. auris* continues to rise across healthcare settings [5]. Together, these pathogens combine antifungal resistance with clinical management complexity and excess mortality [6,7], demanding novel antifungal scaffolds with multi-target activity and the potential to overcome conventional resistance mechanisms. Addressing these challenges demands novel antifungal scaffolds with multi-target activity and the potential to overcome conventional resistance mechanisms.

Against this backdrop, nanotechnology offers new anti-fungal strategies relevant to drug-resistant *Candida*. Among metallic nanomaterials, silver nanoparticles show broad antifungal activity through multiple targets, including membrane and cell wall damage, generation of reactive oxygen species, interference with adhesion and morphogenesis [8,9,10]. Reports and reviews converge on silver nanoparticles as candidates to help prevent or control *C. auris* outbreaks and other difficult to treat candidoses [8]. Within this area, green synthesis can enhance sustainability, biocompatibility, and surface functionality by using biological reductants and capping agents from plants or microbes [10]. Kombucha tea is a fermented infusion produced by a symbiotic culture of bacteria and yeasts that provides a matrix of polyphenols such as catechins, vitamins, and organic acids such as acetic, gluconic, and glucuronic acids, which can reduce and stabilize metal nanoparticles and modulate surface chemistry [11,12]. Beyond the fermentation matrix, components derived from the symbiotic culture can be antimicrobial, and an antibacterial factor identified as 5 hydroxymethylfurfural was recently purified from such extracts [13,14,15]. Yeasts associated with the symbiotic culture, including *Pichia kudriavzevii*, can produce bioflocculants that act as reducing and capping agents for silver nanoparticle synthesis and can yield antimicrobial nanoparticles in a low cost and scalable manner [16]. These observations are consistent with wider green synthesis paradigms in which tea derived phenolics provide electrons and functional groups that direct nucleation and stabilization of silver nanoparticles [17,18]. However, translating these promising physicochemical properties into validated antifungal activity requires biologically relevant efficacy models beyond standard in vitro susceptibility testing.

To connect synthesis with translational readouts, the use of *Galleria mellonella* larvae offers an ethically tractable and temperature tolerant host that survives at 37° Celsius, supports *Candida* infection modeling, enables dose response survival measurements within days, and allows exploration of innate immunity through hemocytes and processes such as phagocytosis and melanization [19]. Methodological reviews have documented the advantages of low cost, simplicity, and a reduced regulatory burden, together with increasing use across fungal pathogens [20]. Outcomes in *G. mellonella* often correlate with mammalian data for antifungal efficacy, which supports its role as a translational bridge between in vitro susceptibility and vertebrate models [21,22].

Despite this convergence of favourable properties, no study to date has combined kombucha-mediated silver nanoparticle synthesis with standardized antifungal evaluation across the full WHO-priority *Candida* panel and simultaneous in vivo validation capturing survival, hemocyte kinetics, and hemocyte viability. In the present study, silver nanoparticles were synthesized using kombucha tea, and anticandidal activity was assessed against a clinically relevant panel that includes *Candidozyma auris*, *C. albicans*, *Nakaseomyces glabrata (C. glabrata)*, *C. dubliniensis*, *C. krusei* (*P. kudriavzevii*), *C. tropicalis*, *C. parapsilosis*, and *C. kefyr*. Standardized in vitro methods were applied to define activity profiles, and efficacy was then evaluated in the *G. mellonella* larva model. This work integrates a sustainable synthesis route with antifungal evaluation across the genus *Candida* and a non-vertebrate in vivo readout that addresses the need for new and biocompatible interventions against high priority *Candida* pathogens, while leveraging kombucha derived reducing and capping chemistries and a model with demonstrated translational relevance.

## 2. Materials and Methods

### 2.1. Materials

The symbiotic culture of bacteria and yeast (SCOBY) starter was purchased from The Kombucha Shop (Kombucha Now, LLC, Madison, WI, USA) [23]. Chun Mee green tea was commercially acquired from Demmers Teehaus (Cluj-Napoca, Romania). Silver nitrate (AgNO_3_), sodium hydroxide (NaOH), methanol, acetic acid, dimethyl sulfoxide (DMSO), sodium citrate, glucose, ethanol, and methylene blue (methylthioninium chloride) were of analytical grade and purchased from Carl Roth GmbH + Co. KG (Karlsruhe, Germany). Cane sugar was obtained from a local commercial supplier. Whatman No. 1 filter paper (Cytiva, Marlborough, MA, USA) and 0.45 µm and 0.22 µm membrane filters (Merck Millipore, Merck KGaA, Darmstadt, Germany) were used for extract clarification and sterilization. Sabouraud dextrose agar, RPMI 1640 medium (with 0.165 M MOPS and 2% glucose), and 3-(4,5-dimethylthiazol-2-yl)-2,5-diphenyltetrazolium bromide (MTT) were used for antifungal susceptibility testing and purchased from Thermo Fisher Scientific (Waltham, MA, USA). Phosphate-buffered saline (PBS) as well as Dulbecco’s phosphate-buffered saline (DPBS, with Ca^2+^ and Mg^2+^) were acquired from Fisher Scientific (Madrid, Spain). Sterile 96-well flat-bottom microtiter plates (Eppendorf SE, Hamburg, Germany) were used for microdilution assays.

Reference strains of *Candida albicans* (ATCC 64548), *Nakaseomyces glabratus* (ATCC 90030), *Candida krusei* (ATCC 6258), *Candida tropicalis* (ATCC 750), *Candida parapsilosis* (ATCC 22019), *Candida dubliniensis* (ATCC MYA-646), and *Candida kefyr* (ATCC 38296) were obtained from the American Type Culture Collection (ATCC, Manassas, VA, USA). A clinical isolate of *Candidozyma auris* (previously *Candida auris*) was obtained from the strain collection of the Department of Microbiology, “Iuliu Hatieganu” University of Medicine and Pharmacy, Cluj-Napoca, Romania. Last-instar *G. mellonella* larvae were purchased from a commercial supplier (nevertebrate.ro, Cluj-Napoca, Romania).

LC-MS/MS analyses were performed on an Agilent 1100 HPLC Series system coupled to an Agilent Ion Trap 1100 SL mass spectrometer (LC/MSD Ion Trap VL, Agilent Technologies, Santa Clara, CA, USA), with separation on a Zorbax SB-C18 column (Agilent Technologies, Santa Clara, CA, USA; 100 mm × 3.0 mm, 3.5 µm particle size). Data acquisition and processing were carried out using DataAnalysis version 5.3 and ChemStation version B01.03 (Agilent Technologies, Santa Clara, CA, USA). Ultraviolet-visible spectroscopy was performed with a Duetta™ Fluorescence and Absorbance Spectrometer (HORIBA Scientific, Kyoto, Japan). Nanoparticle tracking analysis was conducted using a NanoSight NS300 instrument and its proprietary software (Malvern Panalytical, Malvern, UK). Transmission electron microscopy was performed with a Hitachi H-7650 120 kV Automatic TEM (Hitachi, Tokyo, Japan) on carbon-coated copper grids, and particle size measurements were obtained using ImageJ software (version 1.54g, National Institutes of Health, Bethesda, MD, USA). Microplate absorbance readings were recorded using a Multiskan MS microplate reader (Thermo Labsystems, Helsinki, Finland). Inoculations were performed using a Hamilton Gastight 1705 RNR microsyringe (Hamilton Company, Reno, NV, USA). Hemocyte counts were performed using a Thoma counting chamber (depth 0.100 mm, small square area 0.0025 mm^2^; Paul Marienfeld GmbH & Co. KG, Lauda-Königshofen, Germany).

Statistical analyses were performed in GraphPad Prism (version 11.0, GraphPad Software, San Diego, CA, USA), R (version 4.5.1, R Foundation for Statistical Computing, Vienna, Austria) with the survival and survminer packages, and Python (version 3.12) with the scipy, statsmodels, and pingouin libraries.

### 2.2. Kombucha Tea Preparation

Kombucha tea was prepared using a starter culture of SCOBY (Symbiotic Culture of Bacteria and Yeast) obtained from the Kombucha shop (Kombucha Now, LLC, Madison, WI, USA) [23] and Chun Mee tea commercially acquired from Demmers Teehaus in Cluj-Napoca, Romania. Detailed metagenomic characterization of the SCOBY microbial community was not performed, as the present study focuses on the phytochemical profile of the fermented extract as the nanoparticle synthesis substrate, which was characterized longitudinally by LC-MS/MS. First, 1 L of filtered water was brought to a boil and infused with 10 g of organic tea for 5 min. After infusion, the tea was filtered to remove leaves, and 10 g of cane sugar were added and stirred until fully dissolved. The sweetened tea was allowed to cool to 26 °C, after which the SCOBY starter culture and its liquid were introduced. Each container was covered with a breathable cloth secured with a rubber band and maintained at 26 °C in a warm, well-ventilated environment protected from direct sunlight. Fermentation was allowed for 21 days and was considered complete once the pH reached 2.5, indicating sufficient maturity for subsequent nanoparticle synthesis.

### 2.3. HPLC/LC–MS Identification and Quantification of Polyphenolic Compounds in the Kombucha Tea

The phytochemical constituents of the Kombucha tea were characterized at 4 points of time during the fermentation process. A first analysis of the tea was done prior to SCOBY inoculation, then at 7 days fermentation, 14 days fermentation, and finally at 21 days fermentation. The employed technique was realized through LC–MS/MS analysis using two validated protocols. The assays were performed on an Agilent 1100 HPLC Series system linked to an Agilent Ion Trap 1100 SL mass spectrometer (LC/MSD Ion Trap VL) [24,25,26].

The initial protocol aimed to identify and quantify polyphenolic compounds. Separation was conducted on a Zorbax SB-C18 column (100 mm × 3.0 mm, 3.5 μm) with a mobile phase consisting of methanol and 0.1% acetic acid in a binary gradient. Although the method was established for 28 polyphenolic standards, only a portion of these compounds was detected and quantified in the samples. Chromatographic conditions included a column temperature of 48 °C, a flow rate of 1 mL/min, and a 5 μL injection volume. Ultraviolet detection was carried out at 330 nm for phenolic acids and 370 nm for flavonoids, and mass spectrometry was performed in negative electrospray ionization (ESI) mode [27].

A second protocol targeted 8 additional polyphenols—epicatechin, catechin, syringic acid, gallic acid, vanillic acid, protocatechuic acid, epigallocatechin, and epigallocatechin gallate. This method employed the same column and instrumentation but incorporated a modified gradient elution program, with detection in MS mode under the same ESI parameters [27].

Identification of each compound was based on matching retention times and mass spectra to those of authenticated standards. Quantification relied on ultraviolet detection and calibration curves generated from reference standards. Data acquisition and processing were performed using DataAnalysis version 5.3 and ChemStation version B01.03 software (Agilent, Santa Clara, CA, USA). Results were expressed as micrograms of bioactive compound per milliliter of extract.

### 2.4. Biosynthesis and Characterization of Silver Nanoparticles

Kombucha made with Chun Mee tea was chosen to serve as the reducing agent for the biosynthesis of silver nanoparticles (AgNPs). The tea extract was first clarified by filtration through Whatman No. 1 paper until no visible biofilm strands remained. It was then sterilized by sequential filtration through 0.45 µm and 0.22 µm membranes. The sterile filtrate was diluted 1:10 with sterile distilled water to obtain the working solution for nanoparticle synthesis.

For the reduction of silver ions, 50 mL of the Kombucha tea extract were added dropwise to 150 mL of an aqueous AgNO_3_ solution (10^−3^ M) under vigorous stirring. The pH was adjusted to 5 with 1 M NaOH. A rapid color transition from pale yellow to deep red-brown indicated the onset of the bioreduction reaction and the formation of K-AgNPs. After 3 h of reaction, the colloidal suspension was centrifuged at 10,000 rpm for 10 min to collect the nanoparticle pellet, which was washed twice with sterile distilled water [28].

Characterization of the K-AgNPs was performed using complementary spectroscopic and microscopic methods. Ultraviolet–visible spectroscopy was carried out with a Duetta™ Fluorescence and Absorbance Spectrometer (HORIBA Scientific, Kyoto, Japan) across the 300–800 nm range at a 2 nm resolution to confirm nanoparticle formation. Nanoparticle tracking analysis (NTA) was conducted using a Nanosight NS300 instrument (Malvern Panalytica, Malvern, UK) and its proprietary software to determine zeta potential and hydrodynamic size. Particle size distribution and morphology were further examined by transmission electron microscopy with a Hitachi H-7650 120 kV Automatic TEM (Tokyo, Japan) on carbon-coated copper grids. Particle size distribution was determined by manual measurement of 234 particles from five TEM micrographs using Image J software (version 1.54 g).

### 2.5. Antifungal Effects

#### 2.5.1. Determination of Minimum Inhibitory Concentration

The antimicrobial properties of K-AgNPs as well as Kombucha fermented tea were tested on *Candida albicans* (ATCC 64548), *Nakaseomyces glabratus* (ATCC 90030), *Candida krusei* (ATCC 6258), *Candida tropicalis* (ATCC 750), *Candida parapsilosis* (ATCC 22019), *Candida dubliniensis* (ATCC MYA-646), *Candida kefyr* (ATCC 38296), and *Candidozyma auris* (previously *Candida auris*, clinical isolate), by microdilution technique according to EUCAST methodology [29]. Initial fungal suspension was realized by adding five distinct 24 h fungal colonies (grown on Sabouraud dextrose agar at 35 °C, for 24 h) to 3 mL sterile distilled water, adjusting turbidity to 0.5 McFarland. The fungal suspensions were further diluted 1:10 with sterile distilled water until a final inoculum of approximately 1–5 × 10^5^ CFU/mL was achieved. Following the preparation of the fungal suspensions, 100 µL of the standardized fungal inoculum (approximately 1–5 × 10^5^ CFU/mL) was added to the wells of a sterile 96-well microtiter plate.

To test the effects of K-AgNPs and Kombucha tea, serial dilutions of each substance were prepared in RPMI 1640 medium (0.165 M MOPS, 2% Glucose final concentration). For each dilution, 100 µL of the diluted antimicrobial agent was added to the corresponding wells containing the 100 µL fungal suspensions, resulting in final concentrations of: 1/150, 1/200, 1/250, 1/300, 1/350, 1/400, 1/500, 1/750, 1/1000, and 1/1250 (equivalent to 2.66, 2.00, 1.60, 1.33, 1.14, 1.00, 0.80, 0.53, 0.40, and 0.032 µg/mL K-AgNPs). Positive control wells containing only fungal suspensions in RPMI were included to ensure fungal viability, while negative control wells containing only RPMI were used to confirm the sterility. In addition, on every plate, blank background-correction wells containing each K-AgNP dilution in RPMI 1640 without fungal inoculum were prepared in parallel to enable per-concentration subtraction of any optical density contribution from nanoparticle surface plasmon absorbance and light scattering. All analyses were performed quadruplicate to ensure statistical reliability. This control framework, combining growth and sterility controls with quadruplicate testing and independent inoculum verification (described below), was designed to minimize false-positive and false-negative outcomes.

The microtiter plates were covered and incubated at 37 °C for 24 h to allow for fungal growth and interaction with the test substances. After incubation, the plates were examined visually for turbidity and then further assessed by measuring the optical density at 530 nm (OD530) using a microplate reader (Multiskan MS microplate reader, Thermo Labsystems, Helsinki, Finland) to quantify microbial growth. The percent growth for each well was calculated as the OD of the well divided by the OD of the drug-free control, after subtracting the matched per-concentration OD530 measured in the parallel nanoparticle and medium background wells described above. The MIC was defined as the lowest K-AgNP concentration producing ≥80% reduction in optical density at 530 nm relative to the drug-free growth control. This threshold is more stringent than the ≥50% inhibition endpoint specified by the EUCAST reference methods for conventional antifungal agents [29]. The more conservative endpoint was adopted because silver nanoparticle suspensions contribute to background optical density through surface plasmon resonance absorbance and light scattering, which cannot be fully corrected in wells containing both nanoparticles and biological material, making lower inhibition thresholds less reliable for colloidal agents. Each experiment was run in quadruplicate on seven separate days, and results are reported as modal values.

To verify the appropriate inoculum density and ensure accurate MIC determinations, a separate plating step was performed using 50 µL of the standardized fungal inoculum, diluted in 4.95 mL of distilled sterile water. From this dilution, 10 µL was plated onto the Sabouraud dextrose agar plates. The plates were then incubated at 37 °C for 48 h, after which the colonies were counted. The aim was to achieve between 10 and 50 colonies per plate, which corresponds to an appropriate initial inoculum size for the microdilution assay. This step was crucial to confirm that the starting concentration of fungi was consistent across all wells and within the expected range, providing confidence in the reliability of the assay results and the efficacy of the antimicrobial agents tested.

#### 2.5.2. Determination of Minimum Fungicidal Concentration

To determine the Minimal Fungicidal Concentration (MFC) of K-AgNPs and Kombucha tea, an MTT assay was performed following the initial microdilution assay. After determining the MIC and identifying the wells with no visible growth, indicating potential MFC, 50 µL of an MTT solution (0.5 mg/mL) prepared in sterile PBS was added to each well of the microtiter plate. The plate was then covered and incubated at 37 °C for 2 h to allow viable cells to reduce the MTT to formazan, a purple-colored product. Following incubation, 150 µL of DMSO was added to each well to dissolve the formazan crystals. The plate was gently shaken for 10 min to ensure complete dissolution of the crystals. The absorbance of the resulting solution was then measured at 570 nm using a microplate reader. Wells showing no color change, indicating a lack of viable cells capable of reducing MTT, confirmed the MFC identified in the microdilution assay. For the MTT assay, all analyses were performed in quadruplicate to ensure statistical reliability. The absorbance readings were averaged, and the data were analyzed to determine the exact concentrations of K-AgNPs that resulted in no detectable fungal metabolic activity. The MFC was thus defined as the lowest K-AgNP concentration at which no formazan production was detected, indicating the complete absence of metabolically active cells. This metabolic viability endpoint provides a functional confirmation of fungicidal activity that complements the turbidity-based MIC.

### 2.6. In Vivo Experiments Using G. mellonella Larvae

#### 2.6.1. Procurement and Selection of *G. mellonella* Larvae

Larvae were obtained from a commercial supplier in Cluj-Napoca, Cluj County, Romania [30] and transported to the Microbiology Department of “Iuliu Hatieganu” University of Medicine and Pharmacy. All in vivo experiments were initiated within the same week of delivery. Only last-instar larvae weighing 350–450 mg were selected. Individuals showing signs of disease or melanization were excluded from the study. The larvae were maintained at room temperature (24 °C) in sterile plastic Petri dishes containing wood shavings as bedding and kept in darkness until use.

#### 2.6.2. Nanoparticle Toxicity in *G. mellonella* Larvae

The external surface of each larva was disinfected with 70% ethanol prior to injection. A Hamilton Gastight 1705 RNR syringe was used to administer the inoculum through the last left proleg. Each larva received 10 μL of nanoparticle suspension at the following concentrations: undiluted stock, 1/10, 1/100, 1/250, 1/500 or 1/1000 (equivalent to 400, 40, 4, 1.60, 0.80, and 0.40 µg/mL K-AgNPs). Control specimens were injected with 10 μL of Dubelcco’s phosphate-buffered saline using the same procedure. Following inoculation, larvae were incubated at 37 °C for 7 days, and mortality was assessed every 24 h. Death was defined by the absence of movement after tactile stimulation together with dark cuticular pigmentation.

Random assignment to experimental groups was achieved using dedicated randomization software [31]. To control for phenotypic variation among larval batches and to reduce possible seasonal influences on immune responses, experiment replication occurred after a 2-month interval.

#### 2.6.3. *G. mellonella* Fungal Infection Model and Treatment with K-AgNPs

**Figure 1:**
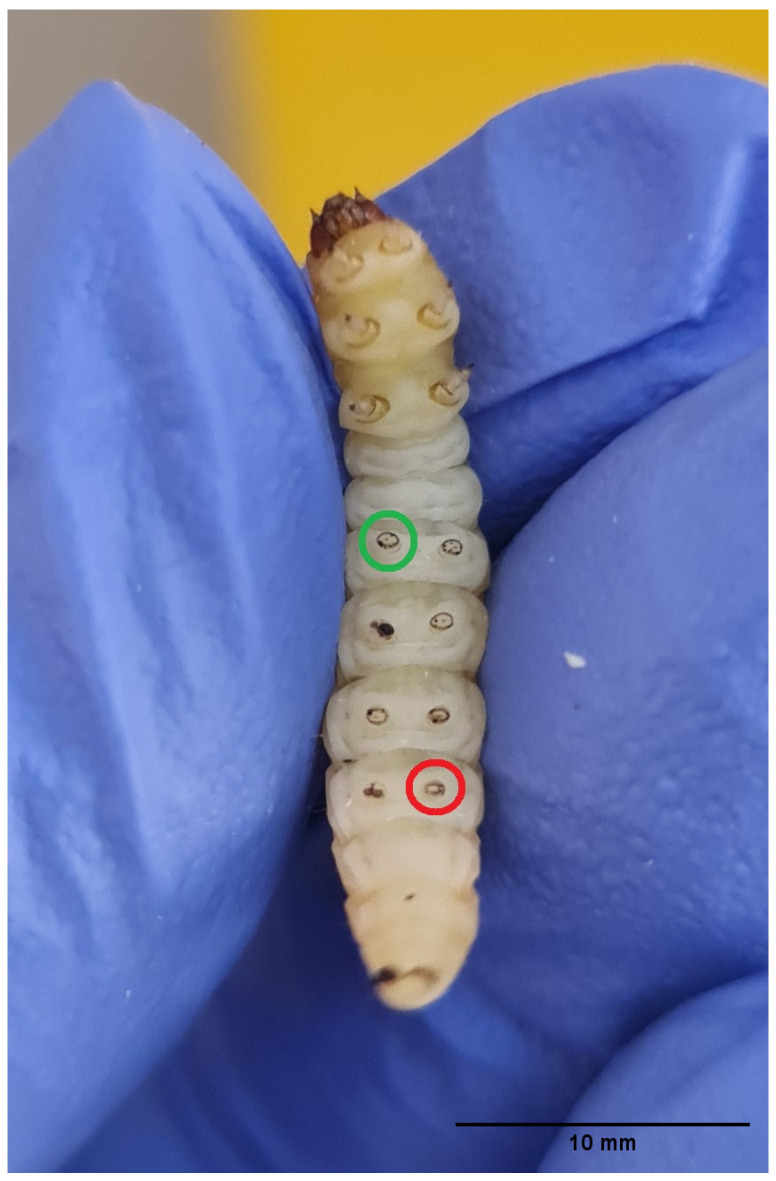
Image depicting injection sites of *G. mellonella* last instar larvae. Green circle—Treatment injection site. Red circle—Fungal inoculum injection site. Scale bar = 10 mm.

Larvae were surface-disinfected with 70% ethanol. Infections were established by intrahaemocoelic injection of a 0.5 McFarland fungal suspension (10 μL) into the last left proleg using a Hamilton Gastight 1705 RNR syringe (Hamilton, Reno, NV, USA). A negative control group received 10 μL of DPBS, while a nanoparticle-only group received 10 μL of species-specific MIC concentrations of K-AgNPs. Each treatment comprised 10 larvae, and the entire experiment was run in triplicate. Larvae were randomly assigned to groups using a random sequence generator [31]. The experiment was repeated two months after the initial run to minimize phenotypic variation between larval batches and potential seasonal effects on larval immunity. Treatments included K-AgNP at the MIC determined for each species (*C. albicans*, *C. glabrata*, *C. krusei*, *C. tropicalis*, *C. parapsilosis*, and *Candidozyma auris*) delivered as 10 μL into the haemocoel via the first proleg with a 10-μL Hamilton microsyringe (Figure 1). Post-injection, larvae were transferred to Petri dishes and incubated at 37 °C, then monitored daily for seven days. Mortality was defined as lack of movement upon tactile stimulation together with darkening of the cuticle. A summary of all experimental groups, species, inocula, and K-AgNP doses across the three *G. mellonella* assays is provided in Supplementary Table S1.

#### 2.6.4. Effects of K-AgNPs on Total Hemocyte Count

Final instar *G. mellonella* larvae (350–450 mg, cream-colored, active, without melanization) were selected for analysis (*n* = 12 larvae per group, per experiment,). *Candida* species were prepared from overnight cultures on Sabouraud dextrose agar, adjusted to a 1.0 McFarland suspension, and subsequently diluted 1:10 in sterile DPBS. Each larva received 10 µL of the inoculum, which was injected into the hemocoel through the last left proleg using a 10 µL Hamilton microsyringe. The species tested included *C. auris*, *C. albicans*, *C. glabrata*, *C. tropicalis*, *C. krusei*, and *C. parapsilosis*. Larvae received K-AgNPs treatment (10 µL) injected into the hemocoel through the first right proleg at the corresponding MIC concentration, as determined in prior susceptibility assays. Four experimental conditions were established: larvae infected with *Candida* spp. but left untreated; larvae infected with *Candida* spp. and subsequently treated with MIC concentration of K-AgNPs; larvae injected with sterile DPBS as a buffer control; and larvae injected with K-AgNPs alone at the MIC concentration. Following injection, larvae were incubated at 37 °C in sterile Petri dishes lined with filter paper and monitored throughout the experiment. At 24 h, 48 h, and 72 h post-injection, hemolymph was collected and immediately processed for total hemocyte count (THC) using a modified DPBS anticoagulant solution and methylene blue viability staining. A higher inoculum density (1.0 McFarland) was used for the hemocyte assay compared to the infection survival model (0.5 McFarland) in order to elicit a more pronounced and temporally compressed immune response. Because hemocyte dynamics were assessed at relatively early time points, a larger fungal burden was judged necessary to ensure that cellular immune parameters would reach measurable and statistically distinguishable levels within this window. This approach was intended to maximize the likelihood of detecting acute hemocyte mobilization and viability changes across experimental groups, without compromising the validity of intergroup comparisons, given that all groups within the hemocyte assay received the same standardized inoculum. Replication of experiment on larvae was realized 2 months after the previous one, in order to eliminate phenotypical differences between different batches of larvae, as well as any seasonal differences in the immune system of larvae.

A modified anticoagulant buffer was formulated using Dulbecco’s phosphate-buffered saline (DPBS; with Ca^2+^ and Mg^2+^) supplemented with compounds possessing anticoagulant and antioxidant activity. The final 200 mL formulation contained:

- Sodium citrate (1.76 g, 30 mM)—To chelate calcium and prevent hemocyte aggregation.
- Glucose (1.8 g, ~0.9% *w*/*v*)—For hemocyte stabilization and mild antioxidant activity.
- Garlic extract (1 mL of a 1:10 dilution)—A natural antioxidant source. The extract was prepared in-house from Romanian garlic (commercial brand, Cluj-Napoca). Bulbs were dehydrated using a household food dryer, ground into powder, and suspended in 12.5% ethanol (10 g garlic powder per 100 mL). The mixture was left to ferment for 60 days at 4 °C, then filter-sterilized (0.22 µm) prior to use.
- Green tea extract (4 mL of a 1:50 dilution)—Providing additional polyphenols with antioxidant capacity. The extract was prepared from Chun Mee green tea (10 g leaves in 1 L distilled water, boiled for 5 min, cooled completely, and filter-sterilized before use).

The buffer was adjusted to pH 7.0–7.2 and stored at 4 °C.

**Figure 2:**
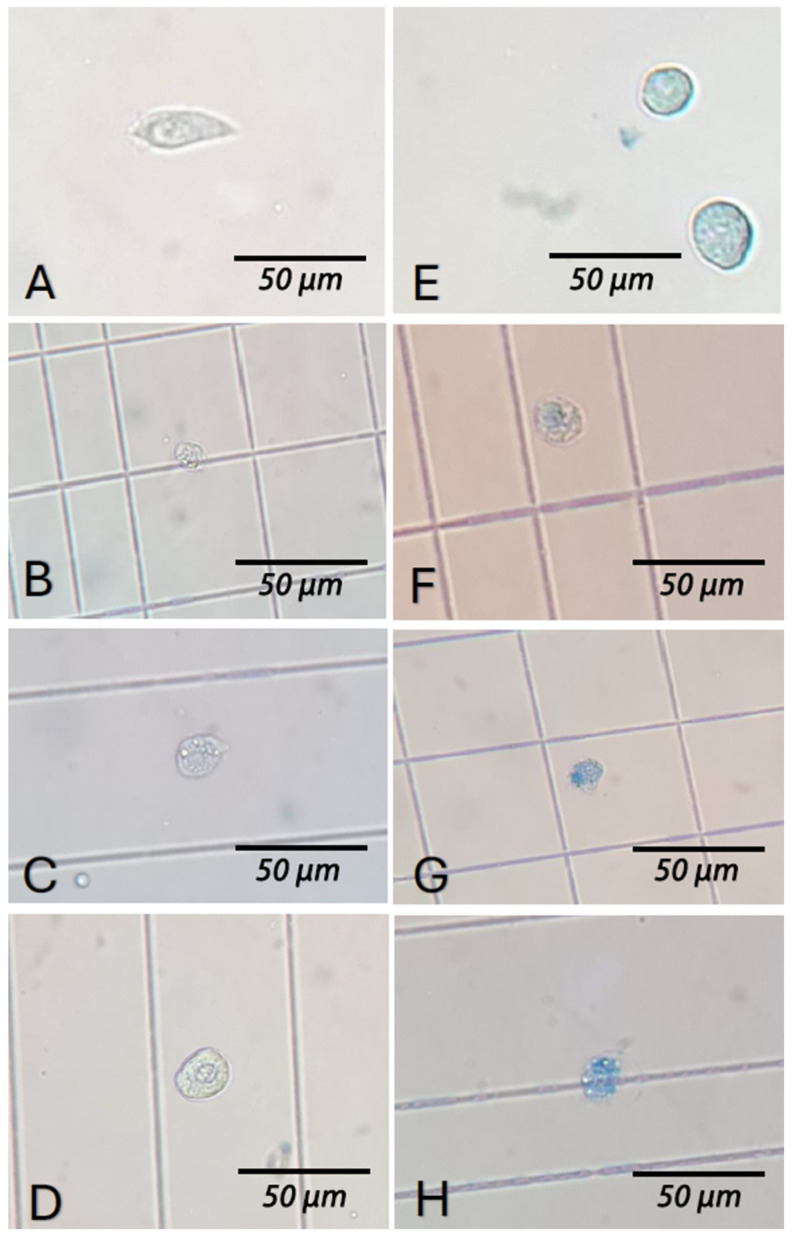
Representative light microscopy images of *G. mellonella* hemocytes assessed by methylene blue dye exclusion viability staining (×400 magnification). Viable hemocytes (**A**–**D**) exclude the dye and appear unstained. Panel (**A**) shows a plasmatocyte, identifiable by its characteristic elongated, fusiform morphology. Panels (**B**,**C**) show granular cells (granulocytes), distinguished by their round to ovoid profile and prominent cytoplasmic granularity. Panel (**D**) shows an oenocytoid, recognizable by its large, round body, distinct plasma membrane, and dense, coiled internal content. Non-viable hemocytes (**E**–**H**) have lost membrane integrity and taken up methylene blue, appearing with blue-stained cytoplasm and nuclei. Panels (**E**,**F**,**H**) show non-viable granular cells at varying degrees of dye uptake, from dense uniform staining (**E**) to partial uptake (**F**,**H**), reflecting different stages of membrane compromise. Panel (**G**) shows a non-viable plasmatocyte retaining its characteristic irregular outline despite advanced membrane compromise. Scale bar = 50 µm.

Prior to hemolymph extraction, larvae were surface-disinfected with 70% ethanol and allowed to dry on sterile filter paper. Hemolymph was collected by decapitation of larvae using sterile scissors. Exuding hemolymph was immediately transferred into sterile tubes containing the modified anticoagulant buffer at a 1:4 ratio (*v*/*v*). Samples were maintained on ice and processed within 1 min to minimize melanization and coagulation. To assess hemocyte viability, methylene blue (methylthioninium chloride, 0.1% *w*/*v*) was prepared directly in the modified DPBS buffer. Equal volumes of diluted hemolymph and methylene blue mixture were mixed (10 µL + 10 µL), incubated at room temperature for 2 min, and loaded into the counting chamber. Under light microscopy, dead hemocytes stained light blue, while viable hemocytes remained unstained (Figure 2).

Cell counts were performed using a Thoma counting chamber (depth 0.100 mm; area 0.0025 mm^2^ per small square). Ten microliters of stained hemolymph suspension were introduced into the chamber and allowed to settle for 1–2 min before observation at ×400 magnification. Hemocytes were counted in 16 large squares, and the total hemocyte count (THC) was calculated according to the formula:

$$THC\;(cells/mL)=\frac{Total\;cell\;scounted\;\times \;Dilution\;factor}{0.1}$$

where 0.1 µL corresponds to the total chamber volume represented by 16 squares. Each sample was counted in duplicate by 2 separate investigators, and results were expressed as mean ± standard deviation.

### 2.7. Statistical Analysis

Total hemocyte count data were analysed using two-way analysis of variance (ANOVA) with treatment group (Infected only, Infected + MIC K-AgNPs, MIC K-AgNPs, and Normal Ctr) and time point (24, 48, and 72 h post-injection) as independent factors, performed separately for each *Candida* species, followed by Tukey’s HSD post hoc tests. Hemocyte viability data were also initially examined by the same two-way ANOVA pipeline; however, normality of residuals (Shapiro–Wilk) and homogeneity of variances (Levene’s test) were violated for the raw viability data across all six *Candida* species owing to a substantial ceiling effect (27.9% of all viability observations at exactly 100%, concentrated in the Normal Ctr and MIC K-AgNPs arms). Four variance-stabilising transformations appropriate to bounded proportion data were evaluated (arcsin-square-root, smoothed logit, reflected square-root, and reflected log) but did not jointly restore both assumptions for five of six species. Hemocyte viability analysis was therefore migrated to a non-parametric framework. The Scheirer–Ray–Hare test was used as the primary two-way analysis with effect sizes reported as epsilon-squared (*ε*^2^), and the Aligned Rank Transform ANOVA was performed as a sensitivity analysis given the known reduction in interaction-detection power of the Scheirer–Ray–Hare test. Pairwise contrasts within each time point were evaluated by Dunn’s test with Holm–Bonferroni correction following an omnibus Kruskal–Wallis test, replacing the Tukey HSD post hoc used for the parametric pipeline. Results are presented as median and interquartile range for hemocyte viability (Tukey box-and-whisker plots, with whiskers extending to the most extreme point within 1.5×IQR of the box and remaining points plotted as outliers) and as mean ± standard deviation for total hemocyte count, with statistical significance indicated on the figures as *p* < 0.05 (*), *p* < 0.01 (**), *p* < 0.001 (***), and *p* < 0.0001 (****). Full ANOVA, non-parametric, and pairwise output, together with diagnostic test results, are provided in Supplementary Tables S2 and S3. All analyses were performed using GraphPad Prism (version 11.0, GraphPad Software, San Diego, CA, USA) for total hemocyte count and R (version 4.5.1) with the rcompanion, ARTool, rstatix, and ggpubr packages for hemocyte viability, with verification in Python (scipy 1.11, statsmodels 0.14).

For survival analysis, daily tallies of living larvae were transformed into individual time-to-event data. Deaths were assigned to the first day observed and larvae surviving to day 7 treated as right-censored. Kaplan–Meier curves were plotted, and group differences were assessed with the log-rank (Mantel–Cox) test. In addition to the overall comparison across groups, pairwise log-rank tests were performed with Holm correction for multiple testing. Analyses and figures were generated in R (v4.5.1) using the survival and survminer packages. Where indicated, plots annotate significance as *p* < 0.05 (*), *p* < 0.01 (**), and *p* < 0.001 (***), based on Holm-adjusted pairwise log-rank *p*-values.

## 3. Results

### 3.1. Kombucha Tea and Biosynthesized K-AgNP Characteristics

LC-MS/MS analysis of Chun Mee kombucha tea identified and quantified 14 of the 22 targeted polyphenolic compounds across the fermentation period (Table 1). The catechin fraction dominated the extract at all time points: epigallocatechin (EGC) was the most abundant compound, ranging from 106,015.7 to 122,631.1 µg/mL, while epigallocatechin gallate (EGCG) was present between 359.3 and 415.8 µg/mL. The most pronounced fermentation-associated change was a progressive ~80% decline in epicatechin, from 5,950.302 µg/mL before inoculation to 1,167.613 µg/mL at 21 days, accompanied by a corresponding progressive increase in gallic acid (36.201 to 46.914 µg/mL) and a rise in protocatechuic acid peaking at 14 days (1.359 µg/mL). Chlorogenic acid and 4-O-caffeoylquinic acid both increased substantially by 7 days and remained elevated relative to pre-inoculation values throughout. The flavonol glycoside fraction—rutoside (rutin), isoquercitrin, and hyperoside—was broadly stable across all time points. Although the 7-day extract (T1) presents the highest total catechin-derived reducing capacity, with EGC and epicatechin at their peak concentrations, the 21-day extract (T3) was selected for nanoparticle synthesis on account of its superior capping chemistry (gallic acid, EGCG, and rutin) which reached their highest concentrations at this time point, providing the richest pool of carboxylate and glycosidic surface-coordinating groups for nanoparticle stabilisation. The pH 2.5 endpoint further ensures that T3 represents a reproducible and well-defined fermentation state, reducing batch-to-batch variability in the synthesis substrate. The spectroscopic implications of the T3 phytochemical profile are discussed in the context of the FTIR analysis below.

**Figure 3:**
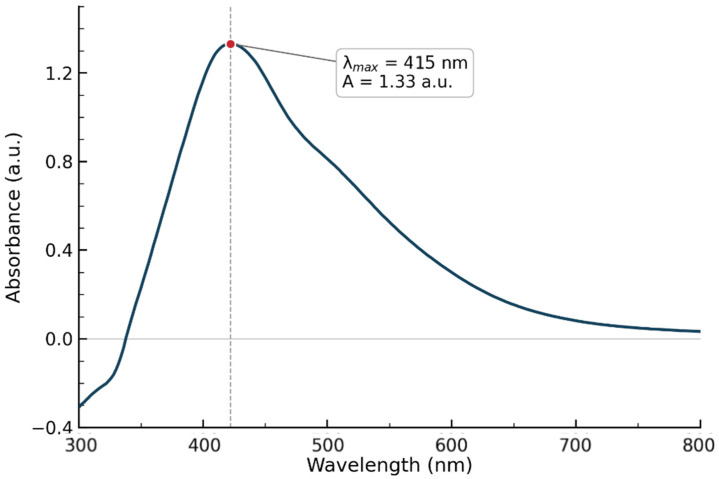
UV-Vis absorption spectrum (300–800 nm) of kombucha-biosynthesized silver nanoparticles solution, showing the characteristic surface plasmon resonance (SPR) peak at 415 nm that confirms the formation of metallic silver nanoparticles.

**Figure 4:**
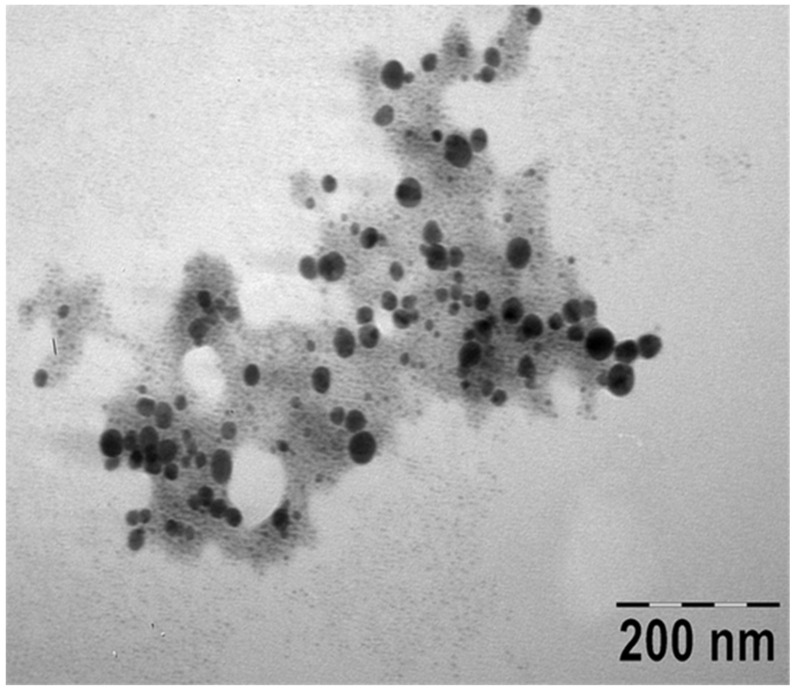
TEM image of the Kombucha biosynthesized silver nanoparticles.

**Figure 5:**
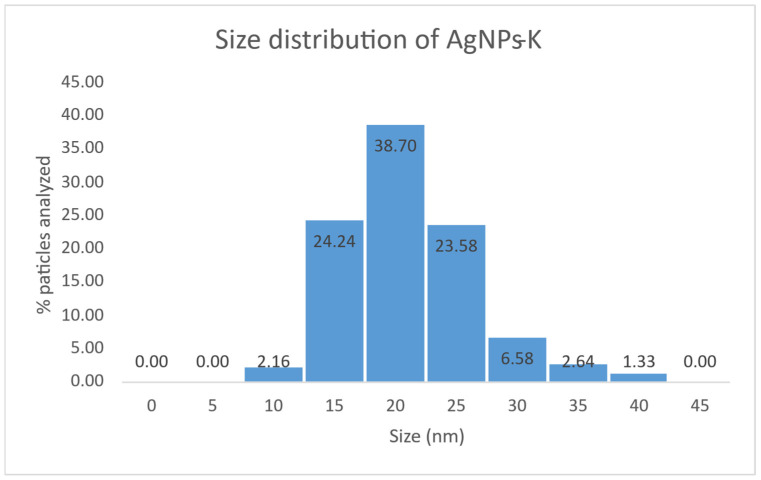
Histogram depicting the size distribution of Kombucha biosynthesized silver nanoparticles (*n* = 234 particles).

UV-Vis spectroscopy confirmed nanoparticle synthesis, with a characteristic absorption peak at 415 nm (Figure 3). Morphological analysis revealed spherical particles (Figure 4), with size distribution analysis showing average size of 19.4 nm (±7.9 nm SD) (Figure 5). NTA measurements indicated a hydrodynamic diameter of 88 nm for the obtained AgNPs-K (accounting for 96.3% of 493 tracked particles), and a zeta potential of −14.52 ± 0.04 mV. The difference between the TEM-derived core diameter and the NTA-derived hydrodynamic diameter is consistent with the presence of the adsorbed polyphenol–glycoside corona and the associated solvation layer in aqueous suspension, a discrepancy routinely observed for surface-functionalized nanoparticles.

**Figure 6:**
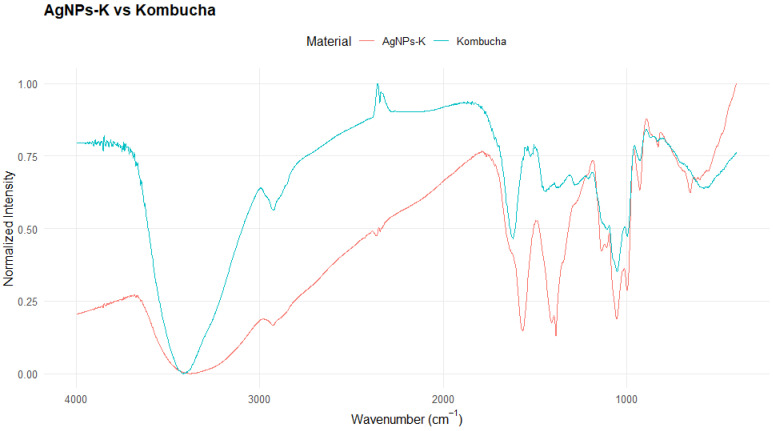
FTIR spectra of Kombucha tea and kombucha mediated AgNPs.

The FTIR spectra of Chun Mee kombucha and the corresponding synthesized K-AgNPs retained a similar overall band profile but showed consistent band shifts and intensity changes after nanoparticle formation, providing direct spectroscopic evidence for capping by kombucha-derived biomolecules and for the involvement of phenolic, flavonoid, and carbohydrate functional groups in Ag^+^ reduction and surface stabilization (Figure 6). The kombucha spectrum displayed a broad O–H stretching band centered at ~3422 cm^−1^ and a C–H stretching band at ~2927 cm^−1^, consistent with the polyhydroxyl-rich architecture of the catechin-class polyphenols identified by LC-MS/MS—in particular EGC and EGCG, which together constitute the dominant chemical mass of the extract. In K-AgNPs, the O–H band shifted to lower wavenumbers (~3385 cm^−1^) with reduced intensity, indicating stronger hydrogen bonding and coordination of phenolic and sugar hydroxyl groups to the silver surface. In the carbonyl and aromatic region, the kombucha spectrum showed a dominant band at ~1621 cm^−1^, while K-AgNPs exhibited a pronounced band at ~1566 cm^−1^ with attenuation of the ~1620 cm^−1^ feature, consistent with deprotonation and coordination of carboxyl and carbonyl groups to Ag^0^. The kombucha bands at ~1444 to ~1427 cm^−1^ shifted and resolved into bands at ~1407 and ~1386 cm^−1^ in the K-AgNPs, a pattern compatible with COO^−^ symmetric stretching of carboxylate groups at a metal surface—a contribution plausibly attributed to the galloyl moiety of EGCG and to gallic acid, which increased progressively across fermentation and was the most abundant low-molecular-weight phenolic acid in the extract at all time points. In the C–O/C–O–C region, kombucha showed bands at ~1107, ~1051, ~995, and ~927 cm^−1^ attributed to glycosidic and polysaccharide vibrations; these were preserved in the K-AgNPs spectrum with slight shifts to ~1135, ~1108, ~1054, ~997, and ~927 cm^−1^ and changes in intensity, supporting adsorption of sugar-rich structures onto the nanoparticle surface. The LC-MS/MS data indicate that the flavonol glycosides—rutin, isoquercitrin, and hyperoside—remained broadly stable across the full fermentation period and are therefore consistently available to contribute this glycosidic capping layer regardless of the fermentation stage from which the extract is drawn.

### 3.2. Antifungal Effects

#### Minimum Inhibitory Concentration and Minimum Fungicidal Concentration

**Figure 7:**
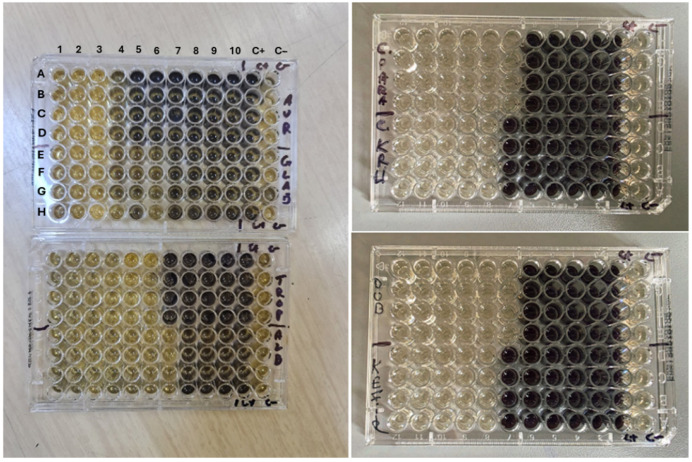
MTT assay results of K-AgNPs-K on tested *Candida* species (realized in quadruplicate). Each plate was used for 2 fungal species. Columns on plates denoting line of replicates (A, B, C, D for one fungal isolate and E, F, G, H for the second). Rows from 1 to 10 contain increasing dilutions of K-AgNPs. Row 11 contained positive control (fungi with culture media), Row 12 sterility control (culture media). Black pigmentation of wells denote active fungal metabolism after application of MTT.

The MIC and MFC were identical for all tested *Candida* species, although the effective dilution varied by species. *C. albicans* showed inhibition and fungicidal activity at 1/500, corresponding to 0.8 µg/mL K-AgNPs. *Candidozyma auris* and *Nakaseomyces glabrata* exhibited overlapping MIC and MFC at 1/250, equivalent to 1.6 µg/mL K-AgNPs. *C. kefyr* and *C. krusei* reached the same endpoint at 1/350, corresponding to 1.14 µg/mL K-AgNPs. *C. tropicalis*, *C. dubliniensis*, and *C. parapsilosis* displayed overlapping MIC and MFC at 1/400, equivalent to 1 µg/mL K-AgNPs. These findings were confirmed by the complete absence of formazan production in the MTT metabolic viability assay at and above the MFC (Figure 7), indicating that no metabolically active cells remained. The concordance between the turbidity-based MIC (≥80% growth inhibition at 530 nm) and the MTT-based MFC (zero metabolic viability) validates that the observed antifungal activity reflects genuine fungal killing rather than optical artefacts attributable to nanoparticle interference.

### 3.3. In Vivo Experiments Using G. mellonella Larvae

#### 3.3.1. Nanoparticle Toxicity in *G. mellonella* Larvae

**Figure 8:**
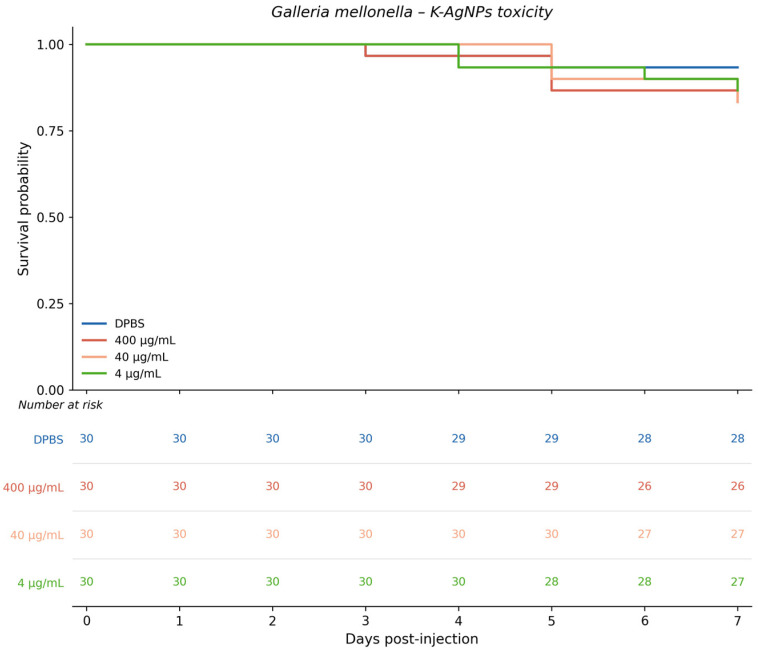
Survival of *G. mellonella* larvae following injection of K-AgNPs across a range of concentrations. DPBS used as control (*n* = 30 per group).

Injection of K-AgNPs across the full concentration range (0.40–400 µg/mL) and a DPBS control (Supplementary Table S1) produced minimal larval mortality over the 7-day observation period, with survival probabilities remaining above 83% in all groups. No statistically significant differences were detected between any K-AgNPs-treated group and the DPBS control, and no dose-dependent decline in survival as observed. Because the near-complete survival across all concentrations produced extensively overlapping Kaplan–Meier curves, a representative subset is displayed in Figure 8 for visual clarity. These findings indicate that K-AgNPs do not exert acute toxic effects in the *G. mellonella* larva model at the concentrations tested, supporting their suitability for subsequent in vivo efficacy experiments.

#### 3.3.2. K-AgNPs Treatment of Fungal Infection Model in *G. molenella* Larvae

**Figure 9:**
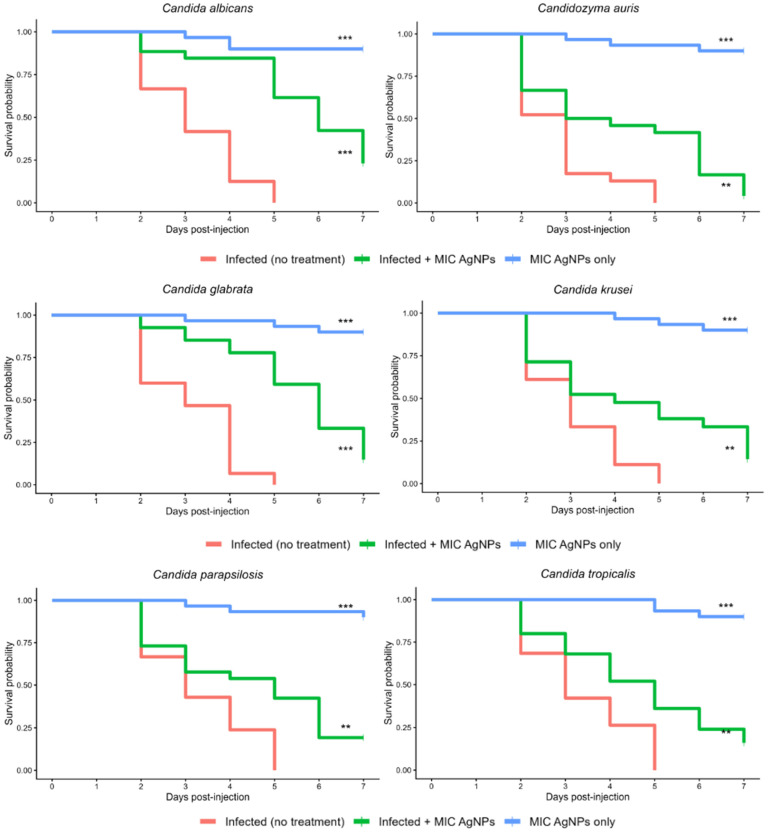
*G. mellonella* larvae survival following infection with *Candida* spp. and K-AgNPs treatment with MIC concentrations for each corresponding fungi (*p* < 0.01 **, *p* < 0.001 ***) (*n* = 30 per group).

Survival analysis across the six yeast infection models demonstrates a consistent pattern, with infection alone inducing substantial larval mortality, whereas exposure to silver nanoparticles at the MIC improved survival in all groups (Figure 9). In every model the AgNPs-only group remained close to 100% survival through day 7 and was significantly higher than the infected, untreated control (*p* < 0.001), confirming an absence of detectable toxicity at the administered dose.

Against *C. albicans*, MIC AgNPs treatment conferred a pronounced survival advantage (*p* < 0.001) relative to infected controls, with most deaths in the untreated arm occurring by days 3–5, while the treated curve declined later and more gradually. A similar magnitude of protection (*p* < 0.001) was observed for *C. glabrata*, where untreated larvae exhibited early step-downs beginning around day 2–3, in contrast to sustained survival under MIC treatment. For *C. auris* and *C. krusei*, MIC treatment produced clear, statistically significant benefits (*p* < 0.01), shifting the onset of mortality to later time points and reducing the overall hazard across the 7-day follow-up. *C. parapsilosis* and *C. tropicalis* also showed a significant treatment effect (*p* < 0.01), with the treated curve separating from the control by day 3 and maintaining a higher survival probability thereafter.

### 3.4. Total Hemocyte Counts

**Figure 10:**
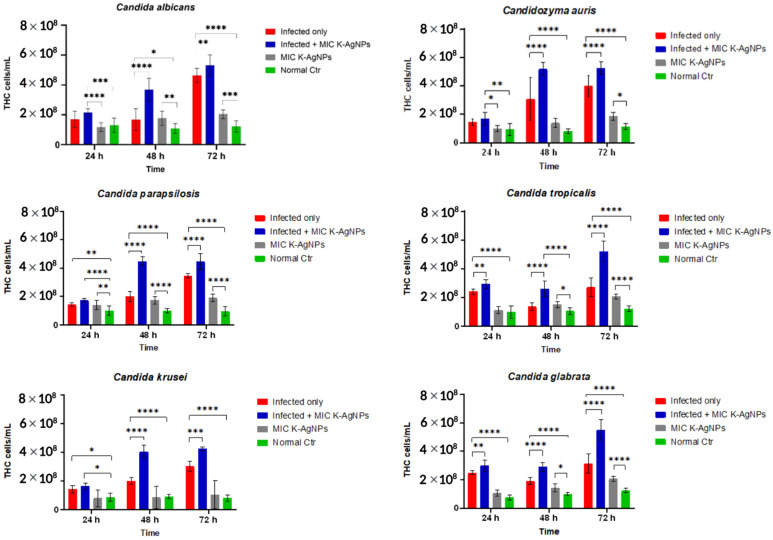
Total Hemocyte Count in *G. mellonella* larvae across four experimental groups at 24, 48, and 72 h post-injection in six *Candida* infection models (*p* < 0.05 *, *p* < 0.01 **, *p* < 0.001 ***, *p* < 0.0001 ****) (*n* = 12 larvae per group per time point).

Across all six *Candida* infection models, total hemocyte count increased over time from 24 to 48 to 72 h, showing strong group-by-time effects and a progressive mobilization of circulating hemocytes. The normal control maintained comparatively low values throughout the experiment, while MIC K-AgNPs alone produced a modest increase that stayed well below the infected conditions. In contrast, infection consistently elevated THC at each time point, and co-treatment with MIC K-AgNPs amplified this response further. The difference between the two infected groups was often smaller at 24 h, but it became clearly pronounced by 48 h and remained evident at 72 h, where infected plus MIC K-AgNPs typically exceeded infection alone with significance levels indicated on the plots, most often in the range of *p* < 0.01 to *p* < 0.0001 (Figure 10).

Although the overall pattern was shared, species-specific differences in hemocyte escalation were still observed. *C. parapsilosis* and *C. glabrata* showed the largest stepwise increases through 72 h, with the co-treatment group reaching the highest late-stage THC. *Candidozyma auris* separated strongly by 48 h and stayed elevated at 72 h, suggesting an early divergence in the systemic response under co-treatment. *C. tropicalis* displayed a later escalation, with the most prominent rise at 72 h. *C. albicans* and *C. krusei* increased more uniformly across the time course, yet still followed the same ordering, co-treatment highest, infection alone intermediate, MIC K-AgNPs alone modest, and normal control lowest.

### 3.5. Hemocyte Viability

**Figure 11:**
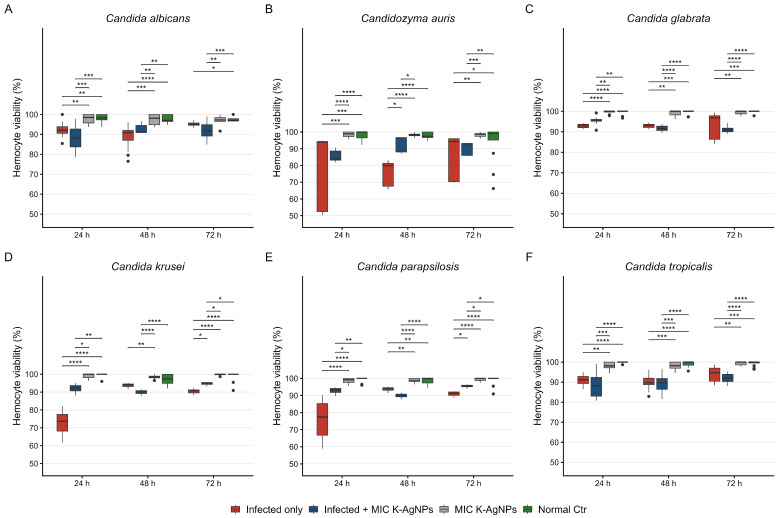
Hemocyte viability (%) in *G. mellonella* larvae across four experimental groups at 24, 48, and 72 h post-injection in six *Candida* infection models. (*n* = 12 larvae per group per time point). (**A**) *Candida albicans*, (**B**) *Candidozyma auris*, (**C**) *Candida glabrata*, (**D**) *Candida krusei*, (**E**) *Candida parapsilosis*, and (**F**) *Candida tropicalis*. Significance is indicated as * *p* < 0.05, ** *p* < 0.01, *** *p* < 0.001, **** *p* < 0.0001. Non-significant comparisons are omitted from the figure for clarity. The MIC K-AgNPs versus Normal Ctr contrast was non-significant at every time point across every species.

Hemocyte viability was assessed at 24, 48, and 72 h post-injection across all four experimental groups in each *Candida* infection model (Figure 11). Because the raw viability data violated the assumptions of normality and homoscedasticity (Shapiro–Wilk and Levene’s *p* < 0.05) and no variance-stabilising transformation jointly restored both assumptions for five of six species owing to a substantial 100% ceiling effect (Section 2.7), the analysis was conducted in a non-parametric framework. The Scheirer–Ray–Hare test confirmed that treatment group was the dominant source of variance in every model (Group main effect, all *p* < 0.0001), with *ε*^2^ values ranging from 0.522 in *C. albicans* to 0.757 in *C. glabrata*, indicating large and consistent effect sizes. A significant Group × Time interaction was identified under the Scheirer–Ray–Hare framework only for *C. krusei* (*p* = 0.015), while under the more powerful Aligned Rank Transform sensitivity analysis the interaction reached significance for five of six species (all except *C. tropicalis*, *p* = 0.059); this divergence reflects the known lower power of the Scheirer–Ray–Hare test for interactions in factorial designs. The *C. tropicalis* interaction was not significant under either framework, indicating a time-stable group structure for that species. Pairwise contrasts within each time point were assessed by Dunn’s test with Holm–Bonferroni correction following Kruskal–Wallis omnibus tests; full output is provided in Supplementary Tables S2 (omnibus analyses with diagnostic tests) and S3 (pairwise comparisons).

Across all six models, larvae in the MIC K-AgNPs group maintained hemocyte viability indistinguishable from Normal Ctr at every time point (Dunn’s test, Holm-adjusted *p* > 0.05), with median values consistently at or above 98%, confirming an absence of hemocytotoxicity at the MIC concentrations employed. Both infected groups generally showed reduced viability relative to the non-infected conditions in every model, though the pattern and timing of this suppression varied substantially by species and the two infected groups were not always statistically separable from each other. In *C. tropicalis*, where no Group × Time interaction was present, the Infected only and Infected + MIC K-AgNPs groups maintained consistently lower viability than MIC K-AgNPs and Normal Ctr throughout (Dunn’s *p* < 0.01 at all three time points for both infected groups versus both non-infected groups), while the two infected groups remained indistinguishable from each other at every time point (*p* > 0.05), suggesting a stable, time-invariant suppression of hemocyte viability by this species. A broadly similar picture was seen in *C. glabrata*, where both infected groups remained significantly below the non-infected groups at all three time points (Infected only and Infected + MIC K-AgNPs each *p* < 0.01 versus MIC K-AgNPs and Normal Ctr at every time point) but were not statistically distinguishable from each other under the non-parametric framework (*p* > 0.05 at all three time points). *C. albicans* produced a more dynamic trajectory, with Infected + MIC K-AgNPs recording the lowest median viability at 24 h (87.8 ± 6.0%), Infected only declining to its nadir at 48 h (88.6 ± 6.1%), and while both partially recovered by 72 h, Infected + MIC K-AgNPs remained significantly below the two non-infected groups at that final time point (*p* ≤ 0.002 versus MIC K-AgNPs and Normal Ctr) without differing significantly from Infected only (*p* > 0.05). The most pronounced early depressions were seen in *C. krusei*, *C. parapsilosis*, and *C. auris*. In *C. krusei*, Infected only viability collapsed to a median around 73% at 24 h, the lowest value recorded across the entire dataset, with all pairwise contrasts versus the two non-infected groups significant at 24 h (*p* < 0.01) but the Infected only versus Infected + MIC K-AgNPs contrast remaining non-significant at 24 and 48 h; partial convergence was seen at 48 h, where Infected only had recovered sufficiently to be statistically indistinguishable from Normal Ctr (*p* > 0.05), before re-separating at 72 h, with all pairwise contrasts again reaching significance except MIC K-AgNPs versus Normal Ctr. *C. parapsilosis* followed a similar arc, with Infected only viability falling sharply at 24 h relative to the non-infected groups (*p* < 0.0001 versus MIC K-AgNPs and Normal Ctr) while remaining non-significantly different from Infected + MIC K-AgNPs, recovering by 48 h, and the two infected groups separating modestly from each other by 72 h (*p* = 0.038). In *C. auris*, Infected only showed the highest variability at 24 h, declined further to its nadir at 48 h, and partially recovered by 72 h, remaining significantly below the non-infected groups at all three time points (Dunn’s *p* < 0.05 versus MIC K-AgNPs and Normal Ctr at every time point); the only time-by-treatment pattern that reached significance under the Scheirer–Ray–Hare framework for this species was the modest separation of the two infected groups at 48 h (*p* = 0.028), while the more pronounced parametric interaction originally reported was not robust to non-parametric re-analysis (see also Section 2.7). In no model and at no time point did Infected + MIC K-AgNPs viability return to levels comparable with MIC K-AgNPs or Normal Ctr.

## 4. Discussion

This study addressed a recognized gap in antifungal therapeutics by integrating an eco-friendly, fermentation-based synthesis approach with standardized in vitro susceptibility testing across a diverse clinical panel and a validated non-vertebrate in vivo model. Taken together, the data demonstrate sub-µg/mL fungicidal activity against all tested *Candida* species, in vivo survival benefit in *G. mellonella* without detectable larvaltoxicity, and a haematologically safe immunostimulatory profile, findings that support further preclinical investigation of kombucha-derived nanomaterials as antifungal candidates.

LC-MS/MS characterization of the Chun Mee kombucha matrix across the full 21-day fermentation revealed a catechin-dominated phytochemical landscape in which EGC (106,015–122,631 µg/mL) and EGCG (359–415 µg/mL) constituted the dominant chemical mass. This catechin dominance is consistent with the known polyphenol architecture of *Camellia sinensis* green tea, where flavanols can represent up to 30% of dry leaf weight [32]. A key fermentation-associated transformation observed in our data was the progressive decline (~80%) in epicatechin, from 5,950 µg/mL pre-inoculation to 1,167 µg/mL at 21 days, accompanied by rising gallic acid (36.2 to 46.9 µg/mL). This pattern reflects a microbially driven hydrolytic biotransformation of ester-linked catechins, a process documented in fermented green tea kombucha wherein EGCG and related gallate esters are hydrolysed by yeast- and bacterial-esterases, liberating epigallocatechin and gallic acid as dominant downstream products [32,33]. Parallel increases in chlorogenic acid and 4-O-caffeoylquinic acid from day 7 onward suggest additional microbial rearrangements of hydroxycinnamic acid glycosides, a pattern also noted in alternative-substrate kombuchas [33]. The flavonol glycoside fraction, consisting in rutin, isoquercitrin, and hyperoside, remained broadly stable across all fermentation time points, consistent with the greater structural resistance of O-glycosylated flavonols to microbial degradation compared with flavan-3-ol monomers [32].

Critically, the selection of the 21-day extract for nanoparticle synthesis was informed by the convergence of high capping-chemistry compounds, with gallic acid, EGCG, and rutin all peaking at 21 days of fermentation, and thus providing the richest pool of carboxylate and glycosidic surface-coordinating groups. While the potent reductants, EGC and epicatechin, were at higher absolute concentrations at 7 days of fermentation, the 21-day profile offers a more favorable balance between reducing capacity and surface-functionalizing ligands for long-term nanoparticle stability. EGCG has been identified as a principal reductant in green tea-mediated AgNP synthesis, with its reduction kinetics a direct function of solution pH [34]. The pH 2.5 endpoint of our 21-day fermentation further standardizes the synthesis substrate, reducing batch-to-batch variability, which provides a methodological advantage rarely reported in kombucha-based nanoparticle synthesis protocols.

K-AgNPs exhibited a UV-Vis SPR peak at 415 nm with the characteristic single, symmetric absorption band associated with spherical metallic silver nanoparticles. This value is well within the 390–470 nm range reported for biogenic AgNPs across a variety of plant extract systems [17,35]. Green tea-mediated AgNPs prepared by Widatalla et al. (2022) [17] displayed an SPR at 410 nm, and those by Kharabi Masooleh et al. (2019) [34], also using EGCG-dominant extracts, resonated between 400 and 435 nm depending on reaction pH, consistent with our observation at 415 nm, given the pH 5 synthesis condition employed. TEM confirmed spherical morphology and a predominant size range of 19.4 nm (±7.9 nm SD), placing K-AgNPs toward the smaller end of the distribution commonly reported for tea-mediated systems (typically 4–75 nm), and within the window associated with maximal membrane penetration and antifungal activity in the literature. Kharabi Masooleh et al. (2019) [34] reported an average hydrodynamic diameter of 10.3 ± 4.6 nm for EGCG-reduced AgNPs synthesized at pH 9, with high negative zeta potential conferring long-term stability. The smaller TEM-derived particle size obtained in our synthesis, such as other polyphenol-capped systems such as CC-AgNPs (~26.89 nm; SPR 438 nm) [36], likely reflects the acidic synthesis pH and the high concentration of multidentate ligands, gallic acid and EGCG, present in the 21-day fermentate, which may kinetically restrict nanoparticle growth by rapid surface saturation during nucleation.

FTIR spectroscopy provided direct mechanistic evidence linking the kombucha phytochemical profile to nanoparticle surface chemistry. The red-shift of the O–H stretching band (~3422 to ~3385 cm^−1^) with reduced intensity in K-AgNPs is consistent with coordination of phenolic hydroxyl groups to the Ag^0^ surface, a signature also described in green tea AgNPs [17]. The emergence of a prominent C=O/C=C band at ~1566 cm^−1^ in K-AgNPs, with attenuation of the 1621 cm^−1^ kombucha band, is indicative of deprotonation and metal coordination of carboxyl groups, while the COO^−^ symmetric stretching pattern at 1407 and 1386 cm^−1^ is characteristic of carboxylate binding at a metal surface, a contribution which can be plausibly assigned to the galloyl moiety of EGCG and to gallic acid [35]. The preservation of C–O–C and glycosidic bands (~1054–1135 cm^−1^) in K-AgNPs shifted from the kombucha reference and confirms adsorption of rutin and other stable flavonol glycosides onto the nanoparticle surface. These FTIR signatures indicate a multi-component, polyphenol–glycoside capping architecture rather than single-ligand surface chemistry, a configuration expected to confer broad colloidal stability and modulate surface charge in a manner correlated with improved biological activity compared to citrate- or protein-capped reference particles [9].

K-AgNPs demonstrated concordant MIC and MFC values across all eight *Candida* species tested, confirming fungicidal rather than merely fungistatic activity at the effective concentration. MIC/MFC values ranged from 0.80 µg/mL (*C. albicans*) to 1.60 µg/mL (*C. auris* and *C. glabrata*), with the remaining species inhibited and killed at 1.00–1.14 µg/mL. These concentrations compare favorably with the broader literature on biogenic AgNPs. AlJindan and AlEraky (2022) [8] reported MIC values of 3.125–200 µg/mL for chemically characterized AgNPs against eight clinical *C. auris* isolates, with IC50 values of 0.7–3.2 µg/mL, against which our MIC of 1.60 µg/mL falls squarely within the most active range. Against *C. albicans*, iturin-capped AgNPs showed a MIC of 1.25 µg/mL at standard inocula [37], while AgNPs from *Pyracantha koidzumii* and *Schinus molle* extracts yielded MIC values of 1.56 µg/mL [38], both closely comparable to our result of 0.80 µg/mL. At the upper end of reported values, polyphenol-capped CC-AgNPs from *Cynara cardunculus* reached MIC and MFC values of 50 and 100 µg/mL against *C. auris* [36], approximately 30-fold above K-AgNP values, a difference attributable to particle size, surface chemistry, and the single clinical isolate versus ATCC strain comparison.

The range review by Mussin and Giusiano (2022) [9], collating MIC data for biogenic AgNPs across fungal species, reported a global range of 0.002–315.5 µg/mL. Our K-AgNPs, with values between 0.80 and 1.60 µg/mL, fall within the lower end of this distribution. For *C. glabrata*, reported MIC for cyanobacteria-derived AgNPs was 25 µg/mL [39], compared with 1.60 µg/mL for K-AgNPs, a 15-fold lower MIC. Similarly, *Alkanna tinctoria*-derived AgNPs showed MIC/MFC of 1.0/2.0 mg/L against *C. parapsilosis* [40], comparable to our value of 1.00 µg/mL; the similar size range (~19.91 nm by TEM) across both studies supports the size-dependence of antifungal activity. Against *C. krusei* and *C. kefyr*, species less frequently evaluated with biosynthesized AgNPs, the present study contributes among the first sub-2 µg/mL MIC/MFC data, suggesting that the complex kombucha-derived surface chemistry confers activity across species with diverse cell wall compositions. The concordance of MIC and MFC values across all species confirms fungicidal activity and is consistent with the multi-target mechanism of AgNPs, which includes cell membrane disruption, ROS generation, and interference with adhesion and morphogenesis [8,9], a mechanistic profile distinct from that of azoles and echinocandins.

To contextualise K-AgNP MIC values against published reference data for established antifungals, EUCAST clinical breakpoints and wild-type MIC distributions provide a useful benchmark. For *C. albicans*, the EUCAST susceptibility breakpoint for fluconazole is ≤2 mg/L and for anidulafungin ≤0.03 mg/L, with wild-type MIC distributions of 0.12–0.5 mg/L and 0.008–0.03 mg/L, respectively [41,42]. The K-AgNP MIC of 0.80 µg/mL against *C. albicans* thus falls within the same order of magnitude as fluconazole activity against susceptible strains, although echinocandins remain more potent on a mass-concentration basis. For *C. glabrata*, which is classified entirely in the “susceptible, increased exposure” category for fluconazole (EUCAST breakpoint R >16 mg/L) [41], the K-AgNP MIC of 1.60 µg/mL represents activity several-fold below the fluconazole resistance threshold. For *C. krusei* (*P. kudriavzevii*), which is intrinsically resistant to fluconazole, the K-AgNP MIC of 1.14 µg/mL demonstrates activity against a species for which azole options are inherently limited. For *C. auris*, which is frequently fluconazole-resistant and for which EUCAST has only recently established echinocandin ECOFFs [43], the K-AgNP MIC of 1.60 µg/mL is notable given the limited therapeutic options available for this pathogen. This comparison is not intended to suggest pharmacological equivalence with conventional antifungals, which have established pharmacokinetic, tissue distribution, and clinical outcome data that K-AgNPs currently lack, but rather to offer contextualisation against published wild-type and breakpoint distributions [41,42,44].

The observation that *C. auris* and *C. glabrata* required slightly higher K-AgNPs concentrations (1.60 µg/mL) than *C. albicans* (0.80 µg/mL) warrants discussion. *C. auris* combines biofilm-forming capacity, thermotolerance, and multidrug resistance [4,5], while *C. glabrata* is known for reduced azole susceptibility linked to CgERG11 upregulation and efflux pumps. Nevertheless, the modest two-fold MIC variation across the entire panel, with even the least susceptible species inhibited and killed at 1.60 µg/mL, is strikingly narrow compared to the large MIC variability seen with conventional antifungals and suggests that the multi-target AgNP mechanism partially circumvents the species-specific resistance determinants that compromise azole and echinocandin activity. Whether cell wall glucan masking in *C. auris* or increased efflux capacity in *C. glabrata* modulates nanoparticle penetration at the subcellular level remains an open question warranting mechanistic investigation.

Survival analysis across six *Candida* infection models demonstrated a consistent and statistically significant protective effect of K-AgNP treatment at the species-specific MIC (*p* < 0.01–0.001 versus infected untreated controls). Equally important, the nanoparticle-only arm maintained near-complete survival through day 7 across all models, providing robust larval safety data at the MIC concentrations used for treatment. In the dose-finding nanoparticle toxicity experiment, larval survival remained above 83% across the full K-AgNP concentration range tested (0.40–400 µg/mL) over the 7-day observation window, with no dose-dependent mortality and no statistically significant difference from the DPBS control at any dose. These findings parallel those of Saberi Moqaddam et al. (2025) [45], who demonstrated that microbially synthesized nano-Ag significantly improved larval survival, increased hemocyte density, and reduced microbial hemolymph load in *C. albicans*-infected *G. mellonella*. Similarly, Thomaz et al. (2020) [46] reported that biogenic AgNPs derived from kefir protected *G. mellonella* against lethal *P. aeruginosa* infection, with approximately 80% prophylactically treated larvae surviving, which presents a broadly similar magnitude of protection to that observed in our *C. albicans* and *C. glabrata* models (*p* < 0.001). The translational relevance of these larval survival outcomes is supported by comparative studies documenting concordance between *G. mellonella* and mammalian antifungal efficacy data [21,22].

The magnitude of survival benefit varied modestly by species. The most significant protection was observed against *C. albicans* and *C. glabrata* (*p* < 0.001), with mortality curves in treated groups declining later and more gradually than in untreated controls. Against *C. auris* and *C. krusei*, treatment produced significant but somewhat attenuated benefit (*p* < 0.01), consistent with slightly higher MIC values and the documented virulence intensity of these species in invertebrate hosts. The survival benefit observed for *C. parapsilosis* and *C. tropicalis* (*p* < 0.01) extends the K-AgNP activity profile to species with increasing clinical importance in candidemia outbreaks and neonatal settings [5]. Notably, the inclusion of *C. dubliniensis* and *C. kefyr* in our panel represents a broader species coverage than most published AgNP antifungal evaluations. Gottardo et al. (2023) [47] evaluated rGO/Ag nanocomposites in *G. mellonella* against *Candida* and dermatophytes and found no nanoparticle toxicity to the larvae, consistent with our AgNP-only survival data; however, their MIC values for *Candida* species ranged from 1.9 to 125 µg/mL, which is considerably higher than those of K-AgNPs. The recent systematic review by Opris et al. (2025) [48] on metal nanoparticle development in *G. mellonella* validated our methodological approach, including triplicate experimentation, two-batch seasonal control, and formal Kaplan–Meier analysis with Holm-corrected pairwise log-rank tests.

The progressive increase in THC from 24 to 72 h across all six infection models, with co-treatment (Infected + MIC K-AgNPs) consistently producing the highest THC at 48 and 72 h, indicates that K-AgNPs do not suppress and may actively potentiate the larval cellular immune response. Thomaz et al. (2020) [46] demonstrated that AgNP-treated *G. mellonella* showed higher circulating hemocyte concentrations than non-treated controls, interpreting this as reflecting pathogen clearance that reduces the hemocyte consumption otherwise associated with uncontrolled infection. Xu et al. (2021) [49] similarly showed that ZnO-NPs increased hemocyte density and activated phenoloxidase in *C. albicans*-infected *G. mellonella*, with immunostimulatory effects identified as a major contributor to in vivo protection, a parallel that raises the hypothesis of a shared nanoparticle-driven immune-priming mechanism across metal oxide and metal nanoparticle systems.

The K-AgNP-only groups produced a modest but consistent THC elevation above normal controls, suggesting that the nanoparticles themselves elicit low-grade hemocyte mobilisation even in the absence of infection, an adjuvant-like priming that may contribute to early fungal containment in the co-treatment arm. The most pronounced progressive THC escalation in co-treated larvae was observed for *C. parapsilosis* and *C. glabrata*, while *C. auris* showed early divergence by 48 h, consistent with the rapid and intense immune activation expected from this highly virulent pathogen. These species-specific kinetics likely reflect qualitative differences in cell wall composition, secreted virulence factors, and immune evasion strategies [19,20]. The *G. mellonella* innate immune system mounts pathogen-specific cellular responses through differential engagement of plasmatocyte and granular cell populations [20], and the THC data capture the systemic intensity of this response at population level across the biologically relevant 24–72 h window.

Hemocyte viability data provided two complementary lines of evidence that together strengthen the overall safety and mechanistic interpretation of K-AgNP co-treatment. Across all six infection models and three time points, the MIC K-AgNPs group maintained viability statistically indistinguishable from the normal control (median values ≥ 98% at all time points, all Dunn’s Holm-adjusted *p* > 0.05), confirming the absence of hemocytotoxicity at the MIC concentrations used for treatment. This finding is particularly reassuring given that AgNPs can induce concentration-dependent cytotoxicity in mammalian cells, with IC50 values for cell viability often in the tens-of-µg/mL range [28]. The sub-2 µg/mL effective concentrations of K-AgNPs appear to sit well below the hemocyte cytotoxic threshold observed in vitro, although mammalian pharmacokinetic and toxicity data will be required to define any true therapeutic window. At the same time, the viability data in the infected groups revealed how each pathogen stresses the hemocyte compartment, adding biological depth to the survival and THC observations already described.

The infection-only groups showed the most pronounced early viability collapse in *C. krusei* (72.9 ± 6.7% at 24 h, the lowest value across the entire dataset), *C. parapsilosis* (76.1 ± 10.9% at 24 h), and *C. auris* (79.9 ± 21.2% at 24 h), all followed by partial recovery at later time points. This trajectory, of acute collapse and recovery, is consistent with intense, rapidly mobilized cytotoxic or immunosuppressive pressure that the host can, at least partly, overcome. An analogous early hemocyte density decline followed by rebound has been described in *C. albicans*-infected *G. mellonella* by Browne et al. (2013) [50], attributed to initial pathogen-driven hemocyte destruction followed by hematopoiesis-mediated replenishment. In contrast, *C. tropicalis* produced a time-stable, non-recovering depression, with the two infected groups indistinguishable from each other across all 72 h, and thus more consistent with sustained immune evasion than with acute cytotoxic attack. Two-way ANOVA confirmed that treatment group was the dominant variance source in all six models (partial η^2^ 0.479–0.839), with the largest effect sizes in *C. krusei* (0.839) and *C. auris* (0.748). The significant Group × Time interaction in five of six models reflects the non-constant character of these differences and underscores the inadequacy of single-time-point viability assessments in multi-species comparative studies. The persistence of Infected + MIC K-AgNPs viability below the non-infected groups at all time points, despite clear survival benefit, indicates that K-AgNPs shift the trajectory of hemocyte stress rather than fully preventing it, reducing the infectious burden and thus modulating the extent of hemocyte damage without fully restoring immune cell health within the 72-h observation window. Future studies extending monitoring to day 5–7 or incorporating phenoloxidase cascade activity and differential hemocyte counts would provide a more mechanistically complete picture of K-AgNP immunomodulatory effects.

To our knowledge, this is the first study to use fermented kombucha tea as the synthesis substrate for AgNPs evaluated against the complete WHO Fungal Priority Pathogens *Candida* panel, including *C. auris*, *C. albicans*, *C. glabrata*, *C. tropicalis*, and *C. parapsilosis*, with in vivo validation that simultaneously captures survival, hemocyte kinetics, and hemocyte viability across six infection models. Tsilo and Pullabhotla (2024) [16] previously used a bioflocculant from *Pichia kudriavzevii* isolated from kombucha SCOBY for AgNP synthesis but did not evaluate antifungal activity against clinically prioritized *Candida* species or perform in vivo testing. The integration of longitudinal LC-MS/MS polyphenol profiling across four fermentation time points with mechanistic FTIR analysis, EUCAST-standardized susceptibility testing, and a multi-parameter in vivo study represents a more holistic characterization than is typically presented in the green synthesis antifungal literature.

Several limitations of the present study should be acknowledged and discussed. First, the study used single ATCC reference strains and one clinical *C. auris* isolate, and since susceptibility can vary substantially across clinical collections, particularly for *C. auris*, expanded testing against geographically diverse multidrug-resistant isolates is necessary. Second, *G. mellonella* lacks an adaptive immune system and cannot model the immunosuppressed clinical scenarios most relevant to invasive candidiasis, meaning that mammalian model data will ultimately be required for translational advancement. Third, the K-AgNP concentration administered in vivo was derived from the in vitro MIC without formal pharmacokinetic characterization in larval hemolymph, and data on nanoparticle distribution, half-life, and tissue accumulation would strengthen mechanistic interpretation. Fourth, the study did not assess antibiofilm activity, which represents a key clinical advantage of AgNPs over conventional antifungals [6,8], and quantitative biofilm inhibition and eradication assays will be addressed in subsequent work.

Fifth, while the characterization suite (TEM, NTA, UV-Vis, FTIR) provides robust physicochemical and surface chemistry data, additional techniques such as X-ray diffraction, yield quantification, and comparison with non-capped AgNPs would further strengthen the synthesis and formulation profile and are planned for ongoing development. Furthermore, in situ physicochemical monitoring of K-AgNP stability under assay conditions, including UV-Vis tracking of the surface plasmon resonance band, dynamic light scattering, and nanoparticle tracking analysis over the 24 h assay window in RPMI 1640, was not performed. Given the relatively high chloride content of RPMI 1640 (approximately 103.5 mmol/L NaCl and 5.3 mmol/L KCl), some Ag^+^/AgCl speciation cannot be excluded, and in-medium nanoparticle stability characterization would clarify the physicochemical state of K-AgNPs during exposure to the fungal inoculum. The internal consistency of the MIC and the metabolic MFC endpoints across all eight species, however, indicates that any such speciation does not abolish biological activity.

Seventh, susceptibility testing did not include parallel comparator arms of conventional antifungal agents such as fluconazole and anidulafungin on the day of testing, with benchmarking being conducted against published EUCAST wild-type MIC distributions and clinical breakpoints rather than against contemporaneously measured comparator MICs, which limits internal validation of assay performance on the day of testing despite the use of EUCAST quality-control reference strains and the EUCAST E.Def 7.4 methodology, and head-to-head MIC determination against licensed antifungals is identified as a priority for follow-up work. OD530 reads were selected to match the visible-light range of microbial turbidity readouts. Although longer-wavelength reading (620–630 nm) would minimise residual plasmon interference even further, the present study mitigated this issue through three complementary measures (adoption of a stringent ≥80% inhibition threshold in place of the EUCAST ≥ 50% threshold, and orthogonal MFC confirmation by MTT metabolic readout), but future MIC determinations of colloidal silver agents are recommended to be performed at 620–630 nm with parallel comparator drug arms. Finally, although methylene blue dye exclusion is the established standard for hemocyte viability assessment in *G. mellonella* [46,51], complementary cytotoxicity and immunophenotyping endpoints, direct in vivo comparison with conventional antifungals, and mammalian toxicity evaluations including erythrocyte haemocompatibility testing will be necessary to build a more complete safety and efficacy profile toward translational development.

Despite these limitations, the present data demonstrate antifungal activity of K-AgNPs across the WHO Fungal Priority Pathogens *Candida* tier, in standardized in vitro and in vivo settings, together with a hemocyte-level safety profile in *G. mellonella* at the concentrations tested. The use of kombucha as a synthesis vehicle adds a further dimension of originality by coupling the well-characterized redox-active chemistry of SCOBY-fermented Chun Mee tea with silver bioreduction, yielding a nanoparticle whose surface is functionalized by a complex naturally derived organic corona that may contribute to antifungal activity independently of the silver core, given the intrinsic antimicrobial properties documented for kombucha-derived polyphenols [12]. These findings, together with the scalability, low reagent cost, and sustainable chemistry of the synthesis route, provide justification for further investigation of K-AgNPs as antifungal candidates, including head-to-head comparisons with licensed antifungals and physicochemical validation of nanoparticle stability under assay conditions.

## 5. Conclusions

The present work demonstrates that a 21-day fermented Chun Mee kombucha extract provides a reproducible and chemically well-defined substrate for the green synthesis of antifungal silver nanoparticles. Longitudinal LC-MS/MS profiling established that gallic acid, epigallocatechin gallate, and rutin reached their highest concentrations at the selected fermentation endpoint, supporting the multi-component polyphenol–glycoside surface corona and linking the synthesis rationale directly to the physicochemical outcome.

Against eight *Candida* species, including the WHO critical–priority pathogen *Candidozyma auris*, K-AgNPs achieved concordant minimum inhibitory and minimum fungicidal concentrations of 0.80 to 1.60 µg/mL, values that fall within the lower MIC range reported for biosynthesized silver nanoparticles in the published literature. The compressed two-fold inter-species MIC range suggests that the multi-target mechanism of silver nanoparticles partially circumvents the resistance architectures that differentiate *Candida* species in their responses to conventional antifungals.

In vivo evaluation in *G. mellonella* showed a significant survival benefit across six infection models without detectable nanoparticle toxicity, while K-AgNPs produced an adjuvant-like hemocyte mobilisation response and maintained hemocyte viability equivalent to negative controls at the concentrations tested. Head-to-head comparison against licensed antifungals, physicochemical validation of K-AgNP stability under assay conditions, antibiofilm evaluation, expanded clinical isolate panels, and mammalian safety assessment, including pharmacokinetic and biodistribution data, will be required before translational claims can be supported.

## Figures and Tables

**Table 1:** Polyphenolic composition of Chun Mee kombucha tea across the fermentation period, determined by LC-MS/MS (concentrations in µg/mL of extract).

Compound	Concentration (µg/mL)			
	T0	T1	T2	T3
Gentisic acid	ND	ND	ND	ND
Caffeic acid	ND	ND	ND	ND
Chlorogenic acid	2.365	3.648	3.497	3.12
4-O caffeoylquinic acid	1.856	3.004	2.927	3.081
p-coumaric acid	ND	ND	ND	ND
Ferulic acid	ND	ND	ND	ND
Vitexin	1.495	2.038	2.581	2.174
Hyperoside	7.452	7.038	7.038	7.763
Vitexin 2-O-Rhamnoside	<LOQ	<LOQ	<LOQ	<LOQ
Isoquercitrin	11.6	11.138	11.138	10.984
Rutoside (Rutin)	14.663	14.366	14.96	15.85
Quercitrin	3.917	2.795	2.235	2.982
Quercetol	0.284	0.339	0.449	0.394
Luteolin	ND	ND	ND	ND
Epicatechin	5950.302	3479.746	2063.986	1167.613
Catechin	0.126	0.18	0.173	0.113
Syringic acid	ND	ND	ND	ND
Gallic acid	36.201	43.823	44.346	46.914
Protocatechuic acid	0.772	1.276	1.359	1.307
Vanillic acid	ND	ND	ND	ND
Epigallocatechin (EGC)	115626.7	122631.1	106015.7	115664.4
Epigallocatechin gallate (EGCG)	386.347	397.232	359.323	415.791

Analyses were performed at four time points: prior to SCOBY inoculation (T0), and at 7 (T1), 14 (T2), and 21 (T3) days of fermentation. Of the 22 compounds targeted, 14 were detected and quantified; 7 were not detected at any time point and 1 was consistently below the limit of quantification. ND = not detected; <LOQ = below the limit of quantification. All values are expressed as µg/mL of extract.

## Data Availability

The original contributions presented in this study are included in the article/Supplementary Material. Further inquiries can be directed to the corresponding authors.

## Supplementary Materials

The following supporting information can be downloaded at: https://www.mdpi.com/article/10.3390/cimb48060634/s1.

## Abbreviations

The following abbreviations are used in this manuscript:

AgNPs	Silver nanoparticles
ATCC	American Type Culture Collection
CC-AgNPs	Cynara cardunculus-derived silver nanoparticles
CFU	Colony-forming units
DMSO	Dimethyl sulfoxide
DPBS	Dulbecco’s phosphate-buffered saline
EC	Epicatechin
ECG	Epicatechin gallate
EGC	Epigallocatechin
EGCG	Epigallocatechin gallate
ESI	Electrospray ionization
EUCAST	European Committee on Antimicrobial Susceptibility Testing
FTIR	Fourier-transform infrared spectroscopy
HPLC	High-performance liquid chromatography
IC50	Half maximal inhibitory concentration
K-AgNPs	Kombucha-derived silver nanoparticles
LC-MS/MS	Liquid chromatography-tandem mass spectrometry
MFC	Minimum fungicidal concentration
MIC	Minimum inhibitory concentration
MOPS	3-(N-morpholino)propanesulfonic acid
MTT	3-(4,5-dimethylthiazol-2-yl)-2,5-diphenyltetrazolium bromide
NTA	Nanoparticle tracking analysis
OD	Optical density
OD530	Optical density at 530 nm
PBS	Phosphate-buffered saline
rGO	Reduced graphene oxide
ROS	Reactive oxygen species
RPMI	Roswell Park Memorial Institute medium
SCOBY	Symbiotic Culture of Bacteria and Yeast
SPR	Surface plasmon resonance
TEM	Transmission electron microscopy
THC	Total hemocyte count
UV-Vis	Ultraviolet-visible spectroscopy
WHO	World Health Organization
ZnO-NPs	Zinc oxide nanoparticles
η^2^p	Partial eta-squared
