# Supplementary material for: Kombucha-Mediated Silver Nanoparticles with Fungicidal Activity Against WHO-Priority Candida Pathogens: In Vitro and Galleria mellonella Evaluation
Source: Curr Issues Mol Biol. 2026 Jun 17;48(6):634. doi: 10.3390/cimb48060634 (PMC13298145; doi:10.3390/cimb48060634)
Supplement: Supplementary file 1 [file cimb-48-00634-s001.zip › Supplementary Table S1.pdf]

Supplementary Table S1. Summary of experimental groups for all *Galleria mellonella* assays. K-AgNP doses in the survival and THC/viability assays correspond to species-specific MIC values determined by EUCAST broth microdilution (Section 3.2). McF, McFarland standard. For the survival assay, *n* represents total larvae across three independent replicates of 10 larvae each, with the entire experiment repeated after a 2-month interval. For the THC and viability assay, *n* = 12 larvae per group per time point (24, 48, and 72 h), sampled by hemolymph collection at each time point.

**Assay**	**Species**	**Inoculum**	**Experimental Group**	**K-AgNP Dose (µg/mL)**	***n***
K-AgNP toxicity	–	–	K-AgNPs 400 µg/mL	400	30
			K-AgNPs 40 µg/mL	40	30
			K-AgNPs 4 µg/mL	4	30
			K-AgNPs 1.60 µg/mL	1.60	30
			K-AgNPs 0.80 µg/mL	0.80	30
			K-AgNPs 0.40 µg/mL	0.40	30
			DPBS control	–	30
Fungal infection model with K-AgNPs treatment	*C. albicans*	0.5 McF	Infected only	–	30
		0.5 McF	Infected + MIC K-AgNPs	0.80	30
		–	MIC K-AgNPs only	0.80	30
		–	DPBS control	–	30
	Candidozyma auris	0.5 McF	Infected only	–	30
		0.5 McF	Infected + MIC K-AgNPs	1.60	30
		–	MIC K-AgNPs only	1.60	30
		–	DPBS control	–	30
	C. glabrata	0.5 McF	Infected only	–	30
		0.5 McF	Infected + MIC K-AgNPs	1.60	30
		–	MIC K-AgNPs only	1.60	30
		–	DPBS control	–	30
	C. krusei	0.5 McF	Infected only	–	30
		0.5 McF	Infected + MIC K-AgNPs	1.14	30
		–	MIC K-AgNPs only	1.14	30
		–	DPBS control	–	30
	C. parapsilosis	0.5 McF	Infected only	–	30

		0.5 McF	Infected + MIC K-AgNPs	1.00	30
		–	MIC K-AgNPs only	1.00	30
		–	DPBS control	–	30
	C. tropicalis	0.5 McF	Infected only	–	30
		0.5 McF	Infected + MIC K-AgNPs	1.00	30
		–	MIC K-AgNPs only	1.00	30
		–	DPBS control	–	30
THC and Viability	*C. albicans*	1.0 McF (1:10)	Infected only	–	12
		1.0 McF (1:10)	Infected + MIC K-AgNPs	0.80	12
		–	MIC K-AgNPs only	0.80	12
		–	Normal Ctr (DPBS)	–	12
	Candidozyma auris	1.0 McF (1:10)	Infected only	–	12
		1.0 McF (1:10)	Infected + MIC K-AgNPs	1.60	12
		–	MIC K-AgNPs only	1.60	12
		–	Normal Ctr (DPBS)	–	12
	C. glabrata	1.0 McF (1:10)	Infected only	–	12
		1.0 McF (1:10)	Infected + MIC K-AgNPs	1.60	12
		–	MIC K-AgNPs only	1.60	12
		–	Normal Ctr (DPBS)	–	12
	C. krusei	1.0 McF (1:10)	Infected only	–	12
		1.0 McF (1:10)	Infected + MIC K-AgNPs	1.14	12
		–	MIC K-AgNPs only	1.14	12
		–	Normal Ctr (DPBS)	–	12
	C. parapsilosis	1.0 McF (1:10)	Infected only	–	12
		1.0 McF (1:10)	Infected + MIC K-AgNPs	1.00	12
		–	MIC K-AgNPs only	1.00	12
		–	Normal Ctr (DPBS)	–	12
	C. tropicalis	1.0 McF (1:10)	Infected only	–	12
		1.0 McF (1:10)	Infected + MIC K-AgNPs	1.00	12

		–	MIC K-AgNPs only	1.00	12
		–	Normal Ctr (DPBS)	–	12

Notes: *Candidozyma auris* is the updated nomenclature for *Candida auris*. A higher inoculum density (1.0 McFarland, diluted 1:10) was used for the THC and viability assay compared to the survival model (0.5 McFarland) to elicit a more pronounced immune response within the 72 h sampling window. The "–" symbol indicates not applicable.
