# Supplementary material for: Kombucha-Mediated Silver Nanoparticles with Fungicidal Activity Against WHO-Priority Candida Pathogens: In Vitro and Galleria mellonella Evaluation
Source: Curr Issues Mol Biol. 2026 Jun 17;48(6):634. doi: 10.3390/cimb48060634 (PMC13298145; doi:10.3390/cimb48060634)
Supplement: Supplementary file 1 [file cimb-48-00634-s001.zip › Supplementary Table S2 REVISED.pdf]

Supplementary Table S2. Non-parametric two-way analyses of hemocyte viability across six *Candida* species. Treatment group (four levels: Infected only, Infected + MIC K-AgNPs, MIC K-AgNPs, Normal Ctr) and time point (three levels: 24, 48, and 72 h) were entered as independent factors. Following the second-round review, the analysis pipeline was migrated to non-parametric methods because both the Shapiro–Wilk and Levene's tests indicated that the assumptions of normality of residuals and homoscedasticity were violated for the raw viability data across all six species, and standard variance-stabilising transformations (arcsin-square-root, logit, reflected square-root, reflected log) did not jointly restore both assumptions for five of six species, largely owing to a substantial ceiling effect (27.9% of all observations at 100% viability, concentrated in the Healthy and MIC K-AgNPs arms). The Scheirer–Ray–Hare (SRH) test is reported as the primary analysis. Because SRH is known to have reduced statistical power for the interaction term in unbalanced or interaction-heavy designs, the Aligned Rank Transform (ART) ANOVA of Wobbrock et al. is reported as a complementary sensitivity analysis with greater power for interactions. Effect size is reported as epsilon-squared (ε^2) for the SRH framework.

Candida albicans							
**Source of variation**	**Scheirer–Ray–Hare (primary)**			**Aligned Rank Transform ANOVA (sensitivity)**			
	**H**	**df**	**p**	**F**	**df**	**p**	**ε^2**
Group	74.255	3	<0.0001	50.272	3,132	<0.0001	0.522
Time	1.110	2	0.574	3.240	2,132	0.042	0.008
Group × Time	6.981	6	0.323	4.955	6,132	<0.001	0.049
Raw data diagnostics — Shapiro–Wilk: W = 0.965, p <0.001; Levene's: F = 4.011, p <0.0001. After arcsin-sqrt transformation — Shapiro–Wilk: W = 0.971, p 0.004; Levene's: F = 1.616, p 0.101.							

Candidozyma auris							
**Source of variation**	**Scheirer–Ray–Hare (primary)**			**Aligned Rank Transform ANOVA (sensitivity)**			
	**H**	**df**	**p**	**F**	**df**	**p**	**ε^2**
Group	84.735	3	<0.0001	79.001	3,132	<0.0001	0.598
Time	0.047	2	0.977	2.835	2,132	0.062	0.000
Group × Time	9.729	6	0.137	5.338	6,132	<0.0001	0.069
Raw data diagnostics — Shapiro–Wilk: W = 0.848, p <0.0001; Levene's: F = 3.103, p <0.001. After arcsin-sqrt transformation — Shapiro–Wilk: W = 0.924, p <0.0001; Levene's: F = 2.100, p 0.024.							

Candida glabrata							
**Source of variation**	**Scheirer–Ray–Hare (primary)**			**Aligned Rank Transform ANOVA (sensitivity)**			
	**H**	**df**	**p**	**F**	**df**	**p**	**ε^2**
Group	102.213	3	<0.0001	146.630	3,132	<0.0001	0.757

Time	1.312	2	0.519	9.301	2,132	<0.001	0.010
Group × Time	5.570	6	0.473	9.505	6,132	<0.0001	0.041

Raw data diagnostics — Shapiro–Wilk: W = 0.815, p <0.0001; Levene's: F = 4.841, p <0.0001. After arcsin-sqrt transformation — Shapiro–Wilk: W = 0.884, p <0.0001; Levene's: F = 2.172, p 0.020.

Candida krusei							
**Source of variation**	**Scheirer–Ray–Hare (primary)**			**Aligned Rank Transform ANOVA (sensitivity)**			
	**H**	**df**	**p**	**F**	**df**	**p**	**ε²**
Group	100.935	3	<0.0001	221.961	3,132	<0.0001	0.727
Time	2.923	2	0.232	65.641	2,132	<0.0001	0.021
Group × Time	15.752	6	0.015	112.414	6,132	<0.0001	0.113

Raw data diagnostics — Shapiro–Wilk: W = 0.851, p <0.0001; Levene's: F = 9.536, p <0.0001. After arcsin-sqrt transformation — Shapiro–Wilk: W = 0.941, p <0.0001; Levene's: F = 2.244, p 0.016.

Candida parapsilosis							
**Source of variation**	**Scheirer–Ray–Hare (primary)**			**Aligned Rank Transform ANOVA (sensitivity)**			
	**H**	**df**	**p**	**F**	**df**	**p**	**ε²**
Group	103.754	3	<0.0001	117.668	3,132	<0.0001	0.750
Time	2.514	2	0.284	49.004	2,132	<0.0001	0.018
Group × Time	12.011	6	0.062	42.553	6,132	<0.0001	0.087

Raw data diagnostics — Shapiro–Wilk: W = 0.701, p <0.0001; Levene's: F = 17.395, p <0.0001. After arcsin-sqrt transformation — Shapiro–Wilk: W = 0.945, p <0.0001; Levene's: F = 3.682, p <0.001.

Candida tropicalis							
**Source of variation**	**Scheirer–Ray–Hare (primary)**			**Aligned Rank Transform ANOVA (sensitivity)**			
	**H**	**df**	**p**	**F**	**df**	**p**	**ε²**
Group	99.657	3	<0.0001	110.043	3,132	<0.0001	0.716
Time	2.586	2	0.274	12.148	2,132	<0.0001	0.019

Group × Time	1.470	6	0.961	2.088	6,132	0.059	0.011
Raw data diagnostics — Shapiro–Wilk: W = 0.956, p <0.001; Levene's: F = 6.002, p <0.0001. After arcsin-sqrt transformation — Shapiro–Wilk: W = 0.982, p 0.049; Levene's: F = 1.298, p 0.232.							

Notes: SRH H, Scheirer–Ray–Hare H statistic, distributed as chi-squared with the indicated degrees of freedom under the null; ε^2, epsilon-squared effect size (SRH framework), interpretable on a 0–1 scale comparably to partial η^2 for parametric ANOVA. ART F-statistics are reported with numerator and residual (denominator) degrees of freedom (n,132). Group main effects retained large effect sizes and were significant under both methods for every species, indicating that the headline biological conclusions of the study are robust to the choice of statistical framework. The Group × Time interaction term is more sensitive to the analytical choice owing to its smaller effect size, this is discussed in Section 4 of the main text.
