# Supplementary material for: Kombucha-Mediated Silver Nanoparticles with Fungicidal Activity Against WHO-Priority Candida Pathogens: In Vitro and Galleria mellonella Evaluation
Source: Curr Issues Mol Biol. 2026 Jun 17;48(6):634. doi: 10.3390/cimb48060634 (PMC13298145; doi:10.3390/cimb48060634)
Supplement: Supplementary file 1 [file cimb-48-00634-s001.zip › Supplementary Table S3.pdf]

Supplementary Table S3. Pairwise Dunn's test (Holm–Bonferroni-adjusted) for hemocyte viability across the four experimental groups at each time point, per *Candida* species. The omnibus Kruskal–Wallis test was performed first within each time point (footer of each species block). Dunn's pairwise contrasts were then computed with Holm–Bonferroni adjustment to control the family-wise Type I error rate across the six pairwise comparisons within each time point. Significance codes: **** p < 0.0001; *** p < 0.001; ** p < 0.01; * p < 0.05; ns = not significant.

Candida albicans						
**Pairwise comparison**	**24 h**		**48 h**		**72 h**	
	**p (Holm)**	**Sig.**	**p (Holm)**	**Sig.**	**p (Holm)**	**Sig.**
Infected only vs Infected + MIC K-AgNPs	0.432	ns	0.683	ns	0.331	ns
Infected only vs MIC K-AgNPs	0.009	**	<0.001	***	0.092	ns
Infected only vs Normal Ctr	0.009	**	<0.0001	****	0.030	*
Infected + MIC K-AgNPs vs MIC K-AgNPs	<0.001	***	0.003	**	0.002	**
Infected + MIC K-AgNPs vs Normal Ctr	<0.001	***	0.003	**	<0.001	***
MIC K-AgNPs vs Normal Ctr	0.982	ns	0.884	ns	0.609	ns
Kruskal–Wallis (omnibus, p)	**<0.0001**		**<0.0001**		**<0.0001**	

Candidozyma auris						
**Pairwise comparison**	**24 h**		**48 h**		**72 h**	
	**p (Holm)**	**Sig.**	**p (Holm)**	**Sig.**	**p (Holm)**	**Sig.**
Infected only vs Infected + MIC K-AgNPs	0.817	ns	0.028	*	0.929	ns
Infected only vs MIC K-AgNPs	<0.001	***	<0.0001	****	0.005	**
Infected only vs Normal Ctr	<0.001	***	<0.0001	****	0.031	*

Infected + MIC K-AgNPs vs MIC K-AgNPs	<0.0001	****	0.028	*	<0.001	***
Infected + MIC K-AgNPs vs Normal Ctr	<0.0001	****	0.076	ns	0.005	**
MIC K-AgNPs vs Normal Ctr	0.883	ns	0.530	ns	0.929	ns
Kruskal–Wallis (omnibus, p)	**<0.0001**		**<0.0001**		**<0.0001**	

Candida glabrata						
**Pairwise comparison**	**24 h**		**48 h**		**72 h**	
	**p (Holm)**	**Sig.**	**p (Holm)**	**Sig.**	**p (Holm)**	**Sig.**
Infected only vs Infected + MIC K-AgNPs	0.142	ns	0.566	ns	0.749	ns
Infected only vs MIC K-AgNPs	<0.0001	****	0.001	**	0.002	**
Infected only vs Normal Ctr	<0.0001	****	<0.001	***	<0.001	***
Infected + MIC K-AgNPs vs MIC K-AgNPs	0.003	**	<0.0001	****	<0.0001	****
Infected + MIC K-AgNPs vs Normal Ctr	0.003	**	<0.0001	****	<0.0001	****
MIC K-AgNPs vs Normal Ctr	0.928	ns	0.655	ns	0.749	ns
Kruskal–Wallis (omnibus, p)	**<0.0001**		**<0.0001**		**<0.0001**	

Candida krusei						
**Pairwise comparison**	**24 h**		**48 h**		**72 h**	
	**p (Holm)**	**Sig.**	**p (Holm)**	**Sig.**	**p (Holm)**	**Sig.**
Infected only vs Infected + MIC K-AgNPs	0.062	ns	0.054	ns	0.041	*
Infected only vs MIC K-AgNPs	<0.0001	****	0.006	**	<0.0001	****
Infected only vs Normal Ctr	<0.0001	****	0.054	ns	<0.0001	****
Infected + MIC K-AgNPs vs MIC K-AgNPs	0.011	*	<0.0001	****	0.012	*
Infected + MIC K-AgNPs vs Normal Ctr	0.001	**	<0.0001	****	0.017	*
MIC K-AgNPs vs Normal Ctr	0.519	ns	0.430	ns	0.833	ns
Kruskal–Wallis (omnibus, p)	**<0.0001**		**<0.0001**		**<0.0001**	

Candida parapsilosis						
**Pairwise comparison**	**24 h**		**48 h**		**72 h**	
	**p (Holm)**	**Sig.**	**p (Holm)**	**Sig.**	**p (Holm)**	**Sig.**
Infected only vs Infected + MIC K-AgNPs	0.070	ns	0.071	ns	0.038	*
Infected only vs MIC K-AgNPs	<0.0001	****	0.006	**	<0.0001	****
Infected only vs Normal Ctr	<0.0001	****	0.006	**	<0.0001	****
Infected + MIC K-AgNPs vs MIC K-AgNPs	0.011	*	<0.0001	****	0.019	*
Infected + MIC K-AgNPs vs Normal Ctr	0.002	**	<0.0001	****	0.022	*

MIC K-AgNPs vs Normal Ctr	0.533	ns	0.977	ns	0.887	ns
Kruskal–Wallis (omnibus, p)	**<0.0001**		**<0.0001**		**<0.0001**	

Candida tropicalis						
**Pairwise comparison**	**24 h**		**48 h**		**72 h**	
	**p (Holm)**	**Sig.**	**p (Holm)**	**Sig.**	**p (Holm)**	**Sig.**
Infected only vs Infected + MIC K-AgNPs	0.630	ns	0.941	ns	0.646	ns
Infected only vs MIC K-AgNPs	0.004	**	<0.001	***	0.002	**
Infected only vs Normal Ctr	<0.0001	****	<0.0001	****	<0.001	***
Infected + MIC K-AgNPs vs MIC K-AgNPs	<0.001	***	<0.001	***	<0.0001	****
Infected + MIC K-AgNPs vs Normal Ctr	<0.0001	****	<0.0001	****	<0.0001	****
MIC K-AgNPs vs Normal Ctr	0.444	ns	0.756	ns	0.853	ns
Kruskal–Wallis (omnibus, p)	**<0.0001**		**<0.0001**		**<0.0001**	

Notes: MIC K-AgNPs vs Normal Ctr is non-significant at every time point across every species, confirming that K-AgNP exposure at the MIC concentration does not reduce hemocyte viability below normal-control levels. Infected only vs Normal Ctr and Infected only vs MIC K-AgNPs are significant at the majority of time points across species, reflecting the species-specific suppression of hemocyte viability by *Candida* infection in untreated larvae.
